# Ambient Carbon-Neutral Ammonia Generation via a Cyclic Microwave Plasma Process

**DOI:** 10.1021/acsami.3c02508

**Published:** 2023-05-03

**Authors:** Sean Brown, Saleh Ahmat Ibrahim, Brandon R. Robinson, Ashley Caiola, Sarojini Tiwari, Yuxin Wang, Debangsu Bhattacharyya, Fanglin Che, Jianli Hu

**Affiliations:** † Department of Chemical and Biomedical Engineering, Benjamin M. Statler College of Engineering and Mineral Resources, West Virginia University, 395 Evansdale Drive, Morgantown, West Virginia 26505, United States; ‡ Department of Chemical Engineering, Francis College of Engineering, University of Massachusetts Lowell, One University Avenue, Lowell, Massachusetts 01854, United States

**Keywords:** ammonia, plasma, hydrogen, chemical looping, catalysis

## Abstract

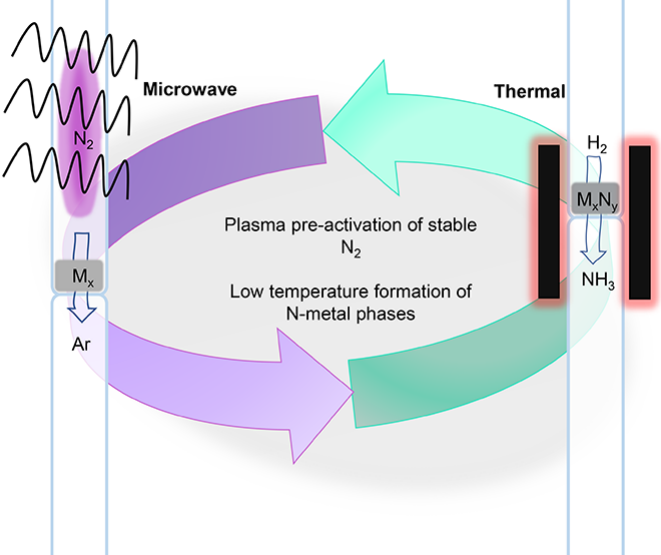

A novel reactor methodology was developed for chemical looping ammonia synthesis processes using microwave plasma for pre-activation of the stable dinitrogen molecule before reaching the catalyst surface. Microwave plasma-enhanced reactions benefit from higher production of activated species, modularity, quick startup, and lower voltage input than competing plasma-catalysis technologies. Simple, economical, and environmentally benign metallic iron catalysts were used in a cyclical atmospheric pressure synthesis of ammonia. Rates of up to 420.9 μmol min^–1^ g^–1^ were observed under mild nitriding conditions. Reaction studies showed that both surface-mediated and bulk-mediated reaction domains were found to exist depending on the time under plasma treatment. The associated density functional theory (DFT) calculations indicated that a higher temperature promoted more nitrogen species in the bulk of iron catalysts but the equilibrium limited the nitrogen converion to ammonia, and vice versa. Generation of vibrationally active N_2_ and, N_2_^+^ ions is associated with lower bulk nitridation temperatures and increased nitrogen contents versus thermal-only systems. Additionally, the kinetics of other transition metal chemical looping ammonia synthesis catalysts (Mn and CoMo) were evaluated by high-resolution time-on-stream kinetic analysis and optical plasma characterization. This study sheds new light on phenomena arising in transient nitrogen storage, kinetics, effect of plasma treatment, apparent activation energies, and rate-limiting reaction steps.

## Introduction

Plasma-enhanced catalysis is an emerging technology that can overcome limitations in traditional heterogeneous catalysis by allowing greater selectivity and productivity through the activation of stable species, surface medication, and generation of vibrationally active species.^1,2^ Plasma catalytic technology combined with new catalyst development may allow low-pressure alternatives to the Haber–Bosch (HB) process.^3^ Ammonia as currently synthesized represents 1–2% of global energy use and 2.5% of global CO_2_ emissions.^4^ As the global population continues to rise and ammonia’s demand increases, opportunities to reduce the energy requirements of the high-pressure (approximately 100 bar) HB process become more attractive.^5^ Similarly, as the world moves closer to the United Nations’ 1.5 °C global temperature, deep emission cuts may require alternative forms of energy storage, such as ammonia as a hydrogen vector.^6,7^ Plasma-enhanced catalytic processes offer the possible benefits of being small-scale, modular, and a part of a renewable energy grid.^8^

**Figure 1:**
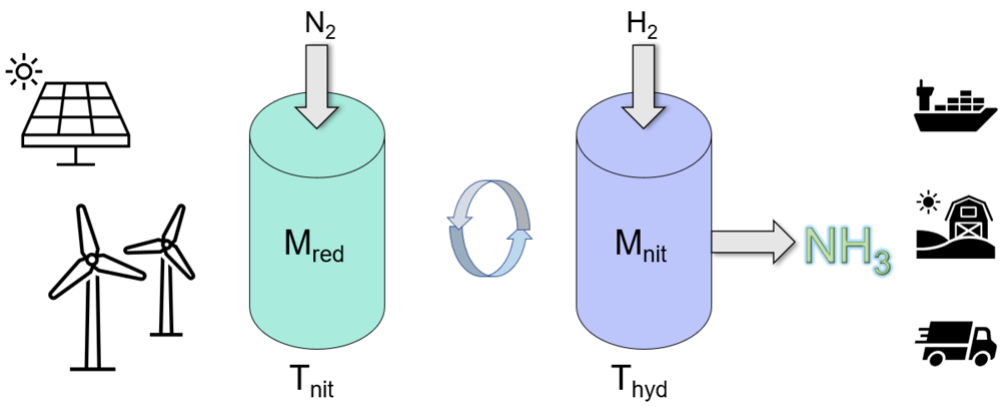
Simplified reaction schematic of the CLAS process. Renewable energy is converted into ammonia in a looped atmospheric process whereby nitrogen reacts in a stepwise manner first to a reduced metal (Mred) and then as a metal nitride (Mnit) to form gaseous ammonia. Both processes occur at different temperatures of nitridation (Tnit) and hydrogenation (Thyd), respectively.

Another strategy to reduce the energy requirement of the HB process is chemical looping ammonia synthesis (CLAS). Figure 1 describes the proposed process.^9^ Ammonia synthesis is a thermodynamically limited reaction, and the CLAS approach separates the N_2_ cleavage in time from the ammonia synthesis step.^10^ These reactions occur on the same material at a low pressure, avoiding the typical limitations placed on industrial HB ammonia synthesis. Several researchers have considered the impact of distributed low-pressure ammonia coupled with renewables and of chemical looping ammonia.^11−13^ Possible energy savings from renewable energy storage in a power-to-ammonia-to-power system yield efficiencies of 38% at time scales greater than 1 day.^11^ While Pfromm and Aframehr suggest that low-pressure approaches have similar energy requirements, they also suggest that process improvements may be made in the form of H_2_ generation and process design, modularization, and simplification for CLAS systems to become more competitive.^12^ Combined ammonia-power generation systems may achieve energy savings.^13^ These analyses are by nature incomplete considering the relative immaturity of the CLAS field more broadly.

Much of the research on CLAS processes has involved advanced material development and proof-of-concept work.^14,15^ Only more recently have researchers considered the thermodynamics and scalability of such processes.^10^ As such, the CLAS approach is in its development stage, and much work remains; however, materials based on nitrogen activity such as Mo and Mn as well as bimetallic alloys and oxide-enhanced or coupled processes with increased rates and contents have been recently published.^9,16−18^

Few studies have evaluated combined plasma catalytic CLAS processes, while the concept of plasma metal nitriding is quite common in the literature. Most recently, Hicks and co-workers published a study of plasma-treated Ni-supported catalysts under temperature-programmed reduction conditions, while not cyclic, this process is very similar to our own.^19^

Finally, most CLAS studies have utilized pH monitoring to determine ammonia productivity over long cycle times.^17^ While simple, cheap, and effective, this method suffers from lack of resolution, which inhibits kinetic data collection and analysis of these complex solid–gas-phase reactions.

Microwave plasma (MWP) reactions with CO_2_ are reported to be very efficient below atmospheric pressure.^1^ However, once these systems reach >0.1 bar, they can achieve a state of local thermal equilibrium.^1^ The exact nature of the thermalization and energy efficiency of the MWP process is dependent on factors of chemistry under consideration and the reactor design.

The N_2_/Ar system never achieves thermodynamic equilibrium between electron and heavy ion temperatures. It may also be assumed that the system is thermalized with the walls of the reactor. Finally, the generation of N_2_^+^ and vibrationally activated N_2_ species is known to greatly enhance the surface reactivity of ammonia synthesis catalysts. Thus, energy efficiency analysis at this stage in process development may not be a useful metric of viability.

In this work, we utilize MWP to pre-treat Fe, Mn, and CoMo CLAS particles before ammonia synthesis under typical thermo-catalytic conditions. These materials are selected because they have a place in the publication record for use as a nitrogen transfer and CLAS material. MWP is also non-equilibrium, but unlike a dielectric barrier discharge plasma (DBD) reactor system, the catalyst bed is placed outside the plasma generation zone, which allows the addition of external thermal heating.^14^ Finally, a kinetic analysis is performed by comparing the post-plasma species with a traditional thermal system to develop more fundamental basic insights on nitride gas-phase reactions.

## Materials and Methods

### Catalyst Materials

CLAS metals were used as received from the manufacturer: Fe (99.9%, <10 μm particle size, Aldrich), Fe nanoparticles (10–30 nm particle size, Thermo Scientific), Mn (99.6%, <10 μm particle size, Alfa Aesar), and Mn nanoparticles (30–50 nm particle size, Alfa Aesar). CoMoO_4_ (99.9%, ∼44 μm particle size, Alfa Aesar) was reduced under 50 sccm H_2_ for 180 min at 750 °C before use in the CLAS process.

### Characterization Methods

Bulk material characterization was undertaken with X-ray diffractometry (XRD), scanning electron microscopy (SEM), and energy-dispersive X-ray spectroscopy (EDX).

XRD was performed with a PANalytical X’Pert Pro PW3040 set to 45 kV and 40 mA that utilizes Cu Kα radiation. Scans were taken from 10 to 100° at a scan rate of 5 °/min.

SEM/EDX was performed with a JEOL JSM-7600F microscope. Imaging was performed at 15.0 kV, with a working distance of 13.4 mm. Elemental mapping was performed at 15.0 kV, with a working distance of 8 mm. The Fe samples were prepared using double-sided carbon tape.

Additional characterization of thermally treated Mn and CoMo samples was performed in our previous publications.^20,21^

### Thermal Fixed-Bed Reactor Experiments

Thermal fixed-bed kinetics were performed using a tubular furnace (Lindberg), mass flow controllers, and quartz tubes to contain the catalyst. A 3/16 in. inlet line to the thermal fixed-bed reactor was used to minimize turbulence, and the outlet line was insulated and heated to the UV–vis inlet. 300 mg of the sample was loaded into quartz reaction tubes (12 mm OD, 8 mm ID, 40.64 cm L) and supported by quartz wool prior to the reaction.

Nitridation reactions were performed under 50 sccm N_2_ (UHP, Airgas) for 1 h at the respective temperatures from the literature: 450 °C Fe and 750 °C CoMo and Mn. The system was allowed to change temperature and purge N_2_ gas under 50 sccm Ar (UHP, Airgas).

Ammonia synthesis reactions were performed under 50 sccm H_2_ (UHP, Matheson) for 30 min at the temperature of consideration. Gas-phase detection of ammonia was performed with a UV–vis ammonia analyzer (Applied Analytics, OMA-406R) collecting concentration data every 11 s. Error bars were calculated using standard error propagation.

### Plasma Fixed-Bed Reactor Experiments

**Figure 2:**
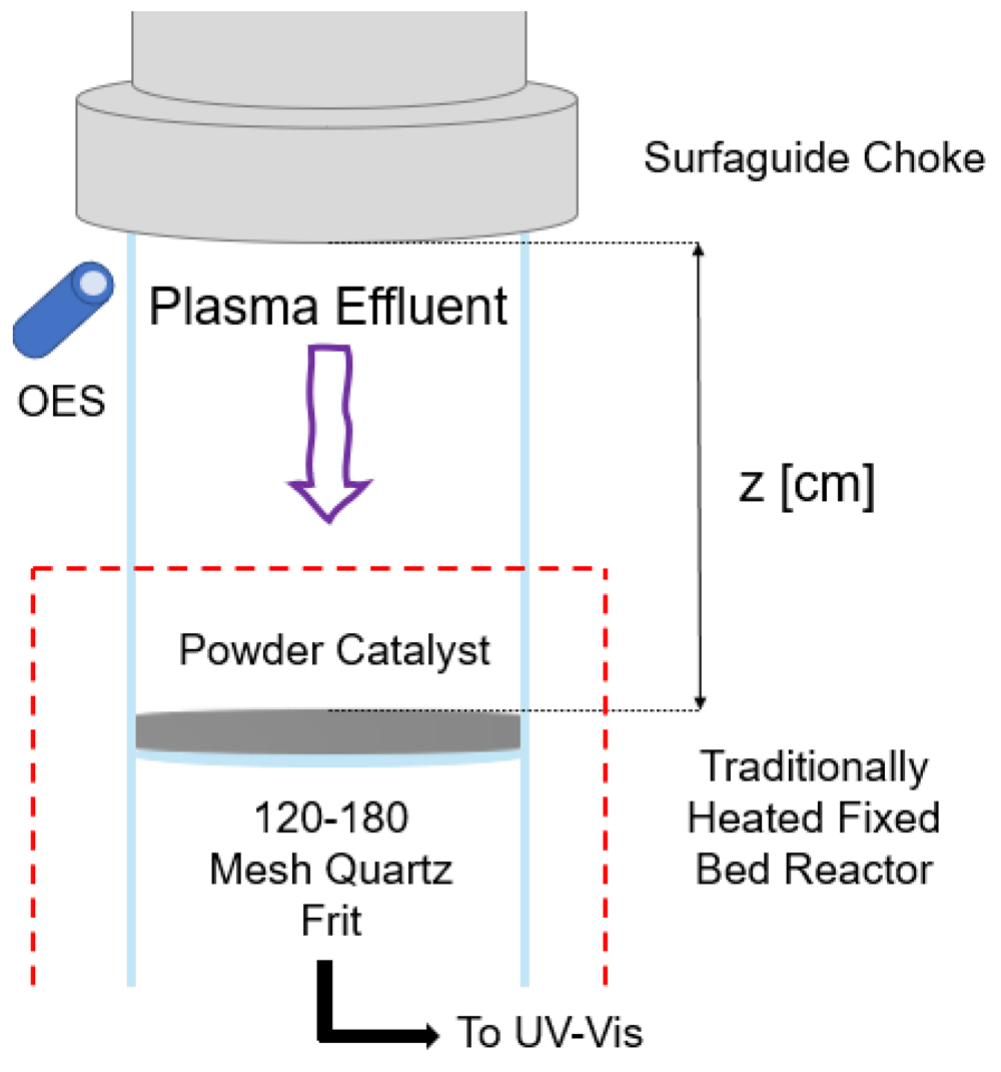
Reactor-catalyst-plasma generating system. A schematic of the idealized plasma-enhanced chemical looping reactor. The outlet of the surfaguide projects plasma in the direction of flow. The catalyst bed sits at *z* ≤ 3 cm between the end of the waveguide and the clamshell furnace. The temperature of the bed is controlled by the tubular furnace. The finely powdered catalyst sits inside a quartz tube in a thin cross-sectional area. Plasma optical emissions are collected by the OES fiber at the outlet of the waveguide choke.

Plasma experiments were performed in the reactor setup (Figure 2), with mass flow controllers and quartz tubes to contain the catalyst (Figure S1). A 3/16 in. diameter inlet line to the thermal fixed-bed reactor was used to minimize turbulence, and the outlet line was insulated and heated to the UV–vis inlet. 300 mg of the sample was loaded into quartz plasma reaction tubes (12 mm OD, 8 mm ID, 61.1 cm L) with a 100–160 μm quartz frit situated 205 mm from the end of the tube, allowing plasma generation above the catalyst which is outside the enclosure of the waveguide choke.

Plasma-enhanced nitridation reactions were performed under 50 sccm N_2_ (UHP, Airgas, Matheson), and a tubular furnace (Mellen) was used to heat the catalyst bed which is located outside the waveguide and beyond the plasma plume in the dark zone. The plasma was turned off, and Ar (UHP, Matheson) gas was used as a purge between steps after the completion of nitridation.

Plasma generation is performed using a 2.54 GHz, 3 kW, fixed-frequency microwave (Sairem, GMP20K). The quartz tube was placed in the waveguide at 300 W power in continuous wave mode, and plasma was ignited using an external spark in pure 50 sccm Ar (UHP, Matheson). The 10 sccm N_2_ (UHP, Airgas) feed was then mixed into the system with 40 sccm Ar balance, and then, a color change was observed from bright blue to deeper purple upon the addition of N_2_.

Ammonia synthesis reactions were performed under 50 sccm H_2_ (UHP, hydrogen) for 15 min at the temperature of consideration. Gas-phase detection of ammonia was performed with a UV–vis ammonia analyzer (Applied Analytics, OMA-406R) collecting concentration data every 11 s.

Optical emission spectroscopy (OES) was used to determine the active species present in the plasma, Ar and Ar/N_2_ mixture, at the end of the waveguide without the catalyst being present. The spectrometer had a spectral range of 200–1100 nm and a 1 nm full width at half-maximum resolution and was supplied with an optical fiber (Ocean Optics, HR 2000_ES). OES emission counts were collected every 18 s.

## Results and Discussion

### Plasma-Reactor Experimental Results

**Figure 3:**
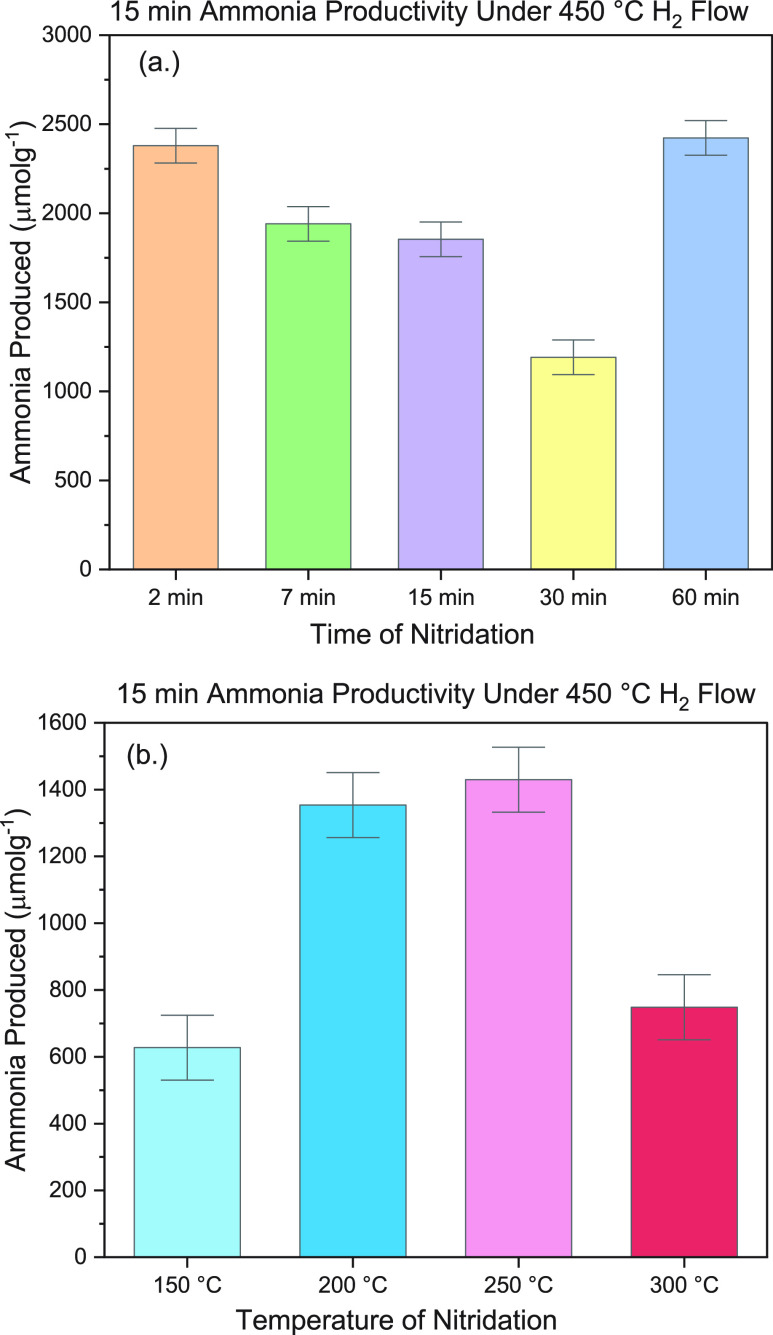
Ammonia productivity under plasma. (a) Impact of plasma nitridation treatment times on productivity was investigated by holding all other variables constant. (b) Optimal temperature of fixed-bed nitridation for Fe particles was investigated by holding all variables constant except for the bed temperature.

Time-on-stream experiments were performed for Fe particles at various nitridation temperatures to determine the productivity increase associated with MWP pre-treatment during the nitridation step (Figure 3a). Changing the temperature of the fixed bed under plasma-nitridation conditions was also investigated as shown in Figure 3b. Productivities were analyzed by integrating time-on-stream concentration results for the ammonia produced from the various nitrided Fe samples over 15 min under flowing 50 sccm H_2_ at the temperature of hydrogenation, 250 °C. High ammonia productivities, 2379 μmol g^–1^, are achieved for short plasma-treatment times at moderate temperatures, 250 °C, with 60 min treatment time only marginally better at 2423 μmol g^–1^.

**Figure 4:**
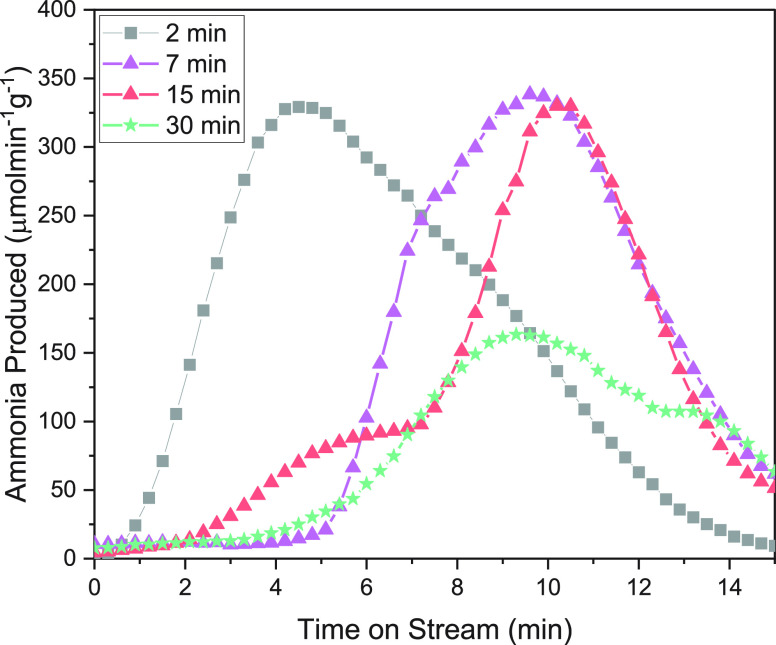
Time-on-stream ammonia productivity. Average time-on-stream reaction results of the hydrogenation of Fe nitrogen carriers under H_2_ flow after being plasma-nitrided for varying time periods.

Fe is known to form various nitride phases under plasma-nitridation conditions.^22^ Iron nitrides have inherently low stability in air; this property coupled with our relatively low bulk conversion was unable to confirm the phases present with XRD or EDX (Figures S2 and S3). Empty reactor tube results yielded no ammonia conversion, so we can surmise that the Fe catalyst is the active site for ammonia synthesis in this reaction. An interesting phenomenon is observed when analyzing Figure 3a and the time-on-stream results in Figure 4. Inspection of the time-on-stream plot in Figure 4 shows a changing shape of the ammonia concentration curve. The shortest plasma-treatment time, 2 min of sustained N_2_ plasma, indicates rapid evolution of ammonia upon the reaction with H_2_. As plasma-nitridation times increase, the productivity was reduced, and the shapes of the curves were observed to change toward a more sigmoid modality.

We propose that this increase in ammonia productivity on a shorter plasma treatment time is due to the effect of competing resistances. A surface-mediated reaction involving only the first several nm of Fe quickly liberates hydrogen. On longer plasma-treatment times, a more bulk-controlled reaction modality is controlling. We observe that with long nitridation times, the initial rate is lower, but the ammonia productivity takes longer as nitrogen diffuses out of the bulk structure to the surface.

Consequently, once the surface becomes saturated with nitrogen and sufficient time has passed, a comparable stage in the reaction time is reached through diffusion. Comparing the initial rates of each nitridation time supports this interpretation (Figure S4). A more thorough development of this effect is considered in the mechanistic section.

The plasma reaction order was determined via power-law kinetics to be 0.72 (Figure S4). The rates and kinetic parameters were determined via the shrinking core model (SCM), as shown in Table 1 (Figures S6 and S7). The rates obtained indicate a comparable production of ammonia from the lower-temperature MWP pre-treatment process to a traditional thermochemical route. The SCM kinetics were used to determine the apparent activation energies, (*E*_a_app__), of the Fe process, 13 and 20.6 kJ mol^–1^ for plasma and thermal treatments, respectively (Figure S8). Typically, if a reaction is known to follow one limiting case, such as bulk diffusion, then the construction of the activation energy may differ greatly depending on the nature of the reacting system. In this case, we have selected to analyze a simple “apparent” energy that does not have the granularity to discern each step of the reaction process’ relevant activation energies.^23^

**Table 1:** Plasma-Treated Particle Kinetic Analysis^a^

temperature of nitridation [°C]	rate [μmol g^–1^ min^–1^]	apparent rate constant [s^–1^]	temperature of hydrogenation [°C]	rate-determining step	flow rate [sccm]	time on stream [min]	*R*^2^
150	130.5	1.05 × 10^–3^	450	gas	50	16.8	0.9939
200	304.3	1.43 × 10^–3^	450	surface	50	10.5	0.9436
250	420.9	5.07 × 10^–4^	450	gas†	50	29.7	0.9267
300	150.9	9.07 × 10^–4^	450	gas	50	17.4	0.9427
450	505	7.70 × 10^–2^^b^	450	n.a.	50	49	0.9187

a Assumed first-order reaction to obtain initial rates of ammonia synthesis on plasma-treated Fe particles.

b Determined by the power-law kinetic method, resulting in a reaction order of *n* = 0.72.

Several experimental fits of the SCM suggested multiple reaction-limiting steps as ammonia synthesis proceeded. SCM fits were calculated individually for each reaction step, not globally optimized; however, this method is the typical one used in the CLAS, not the chemical looping combustion (CLC) literature.^24,25^

Time-on-stream experiments were performed for Fe, Mn, and CoMo particles at various hydrogenation temperatures to determine the reaction rates and apparent activation energies assuming an Arrenhius relationship. Additional time-on-stream experiments were performed with varying particle sizes and varying flow rates to determine mass transfer effects on the ammonia synthesis reaction rate. The time of the reaction was limited to the initial reaction kinetics, and a time step for hydrogenation was chosen to be 15 min.

Typically, such reactions are considered in an SCM for particles with unchanging size.^25,26^ Applying the SCM framework allows the determination of limiting regimes, gas diffusion, surface reaction, and bulk diffusion, in lieu of more detailed elementary step analysis. However, the literature on the hydrogenation of nitrides also suffers from a lack of repeated time-on-stream studies, instead of relying on pH metering during extended reaction times.^27−29^ While being useful to calculate conversion, this kind of data may smooth over process dynamics, which exist in real reacting particle regimes. Recent literature on applying the SCM highlights difficulties in blindly applying the mode without fundamental consideration of the reacting system.^24^ It is our intention to rectify the lack of high-resolution time-on-stream data, and future work will address the limitations inherent in using the SCM.

Results for the kinetic analysis of thermal fixed-bed hydrogenation reactions of CoMo, Mn, and Fe samples are presented in Table 2. These results may be compared with similar studies published elsewhere, CoMo, Fe, and Mn, for instance, were found to have initial rates of ∼98 μmol h^–1^ g^–1^ (400 °C, 1/3 Ar/H_2_, 60 mL min^–1^, 0.4 g), ∼50 μmol h^–1^ g^–1^ (400 °C, 1/3 Ar/H_2_, 60 mL min^–1^, 0.3 g), and ∼635 μmol h^–1^ g^–1^ (500 °C, H_2_O, 0.1 mL min^–1^, 0.5 g), respectively.^25,27,29^ Time-on-stream plots, SCM equations, and fitted lines can be found in the Supporting Information. Many of the lines of best fit suggest that multiple reaction schemes may be controlling but lack fundamental information on the reaction, and we instead rely on the SCM. The model selected is the one that best fits the reaction.

**Table 2:** Thermally Treated Particle Kinetic Analysis^a^^,^^b^

material	rate [μmol g^–1^ min^–1^]	temperature [°C]	*k* [s^–1^]	rate-limiting step	flow rate [sccm]	time on stream [min]	*R*^2^
CoMo	45.6	350	2.8 × 10^–4^	gas	50	60	0.9998
	62.4	450	4.47 × 10^–4^	gas	25	15	0.9387
	31.2	450	1.21 × 10^–3^	surface	50	16.5	0.9950
	180.0	450	4.37 × 10^–4^	gas	100	30	0.9321
	66.7	550	4.15 × 10^–4^	gas	50	33	0.9821
Mn	64.4	300	4.92 × 10^–4^	gas^c^	50	30	0.9839
	41.1	350	5.01 × 10^–4^	gas^c^	50	30	0.9871
	191.3	450	3.10 × 10^–4^	gas^c^	50	44.1	0.9559
	261.9	450	2.56 × 10^–4^	gas^c^	25	60	0.9935
	211.3	450	1.31 × 10^–3^	gas^c^	100	12.3	0.9977
	306.1	500	4.29 × 10^–4^	gas	50	31.4	0.9603
Mn np	29.1	350	1.99 × 10^–3^	gas	50	9	0.9865
	689.1	450	1.93 × 10^–4^	gas^c^	50	60.3	0.8952
Fe	312.9	250	7.57 × 10^–4^	gas	50	15.3	0.8614
	606	350	9.88 × 10^–4^	gas	50	16.2	0.9512
	362.2	400	7.09 × 10^–4^	surface	50	22.2	0.9222
	61.9	450	4.81 × 10^–4^	gas	25	28.2	0.9494
	588.7	450	1.93 × 10^–3^	surface	50	9	0.9804
	388	550	3.02 × 10^–4^	gas	50	11.4	0.9678
Fe np	100	250	3.24 × 10^–4^	gas	50	47.4	0.9812
	110.7	350	7.13 × 10^–4^	gas	50	18.6	0.9376
	92.7	450	4.79 × 10^–4^	gas	50	35.1	0.9923
	187.3	550	4.79 × 10^–4^	gas	50	69	0.9479

a Thermal-only fixed-bed kinetics determined by the shrinking core reaction model for spheres of constant volume.

b Shrinking core conversions (X) determined by the maximum conversion after integration of the concentration across an experiment.

c The model selected had the highest *R*^2^ value, but the other rate-determining steps, bulk diffusion, and surface reaction were also found to be highly significant.

### Plasma Characterization

To understand the plasma system, input variables such as power, frequency, flowrate, and composition were considered. Additionally, emission spectra were collected via an OES optical fiber from the plasma plume. The optimal plasma composition and flow were found to be at 300 kW input power and 40 sccm Ar and 10 sccm N_2_; additional compositions and flows were tried. A review of the literature suggests that an optimum exists for MWP with a 20% N_2_ and 80% Ar composition for the formation of activated nitrogen in the plasma.^30,31^

**Figure 5:**
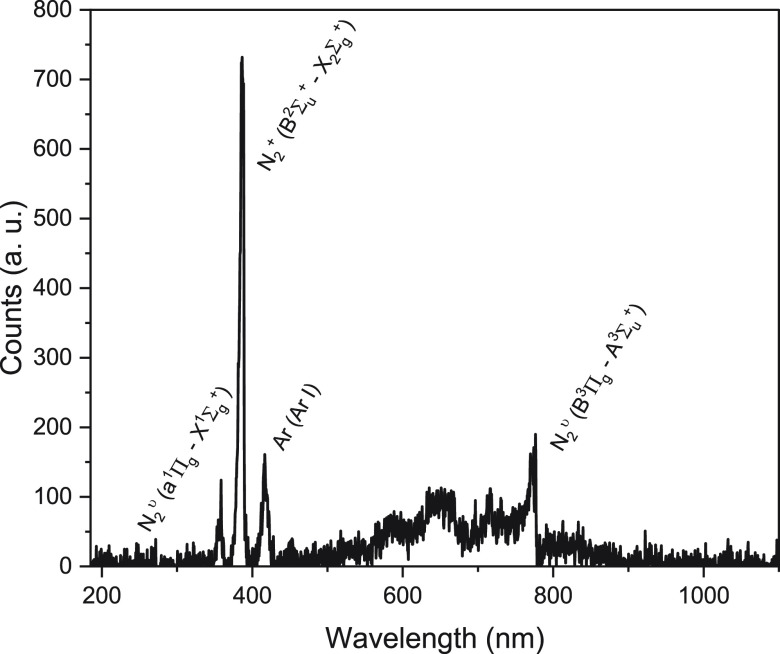
Optical emission spectra collected for the plasma. The optical emission spectra collected from the outlet choke of the surfaguide waveguide (10 sccm N_2_ balanced in 40 sccm Ar, 300 W).

Spectra were obtained from both the center of the plasma plume from a port in the surfaguide and from the choke at the outlet to the tubular furnace. While not truly “in situ” spectroscopy, the placement of the OES allows observation of the plasma <3 cm before effluent gases reach the catalyst surface. Fortunately, the lifetimes of activated species may be calculated by using the electron temperature, ∼5500 K, at the end of the plume, which depends on an unknown function of the length *z* (Figure S9). With this information from the analysis of the spectra collected in Figure 5 using the Boltzmann plot method, flowrates, and basic geometry of the system, the species reaching the Fe catalyst surface may be inferred.

Important considerations for nonthermal or non-equilibrium MWPs are that the electron temperature (*T*_e_) is higher than the gas temperature (*T*_g_) (*T*_e_ > > *T*_g_). MWPs typically have an electron density (*n*_e_) of 10^20^–10^24^ m^–3^ and a gas temperature (*T*_g_) that is ∼2000–3000 K in the plasma zone.^32,33^ This typically results in a non-equilibrium plasma system, which maintains neutrality and has considerably hotter electrons than ions, atoms, and molecules. Testing in our MWP reactor with a thermal couple in the catalyst bed only resulted in slightly elevated gas temperatures as compared to ambient temperature, meaning that most of the thermal energy is conserved in the plasma discharge region. By analyzing molecular and atomic spectra for the Ar/N_2_ system, we can assign some of the peaks in Figure 5 to the species expected in an MWP discharge. These include the first negative system (FNS) of N_2_^+^ (388 and 391 nm) and the activated vibrational states of N_2_ (358 and 776 nm) and Ar I (417 nm), along with lesser peaks in the range of neutral Ar I and N_2_^v^.^34−37^

The possible reactions between Ar and N_2_ in the plasma are many, and more information may be extracted by inspecting the Ar spectra collected from the center of the plume (Figure S10), but N_2_^+^ and the two N_2_ vibrational states are the major products. A common reaction is the charge transfer one, where Ar is easily ionized to Ar^+^ and heavy ion collisions occur with N_2_, generating activated N_2_^+^ and other species.^34^

Detailed calculations may be found in the Supporting Information. Lesser-intensity Ar I emissions are grouped between 696 and ∼800 nm.^35,37^ Vibrationally active N_2_ and other species of N_2_^+^ may persist for between ms and 10 s and may reach the catalyst bed, especially considering the energy distribution of plasma systems.^38^

This topic has become increasingly relevant, with publications suggesting that under plasma conditions, vibrationally active species interact differently with surfaces, altering bond energies.^2,39^

To rule out a simple thermal increase in the system due to MWP, a thermocouple was inserted where the catalyst typically sits during normal operation. Several runs under the Ar/N_2_ plasma condition revealed only a small ∼5 °C temperature change. This is supported by experimental evidence, which found that elevated MWP Ar/N_2_ temperatures (3000 K) return to normal (400 K) only 3 cm outside the plasma zone.^31^

### Proposed Mechanism

A major drawback of most SCM models applied to chemical looping combustion is the complexity of reactions; even with a “simple” system such as the oxidation of Fe particles, accurately modeling them can become both difficult and require modification of the original model.^24^ Kinetic parameters determined from overfitting can result in loss of data and incorrect assumptions of rate-limiting steps as critical kinetic steps are overlooked.^24^

In our system, this is complicated by the inclusion of the Ar/N_2_ MWP reactions, which result in many possible reactions that depend upon plasma conditions. Modeling of the catalyst system under the plasma condition is further complicated by charge accumulation.^40,41^ Increased ammonia production was observed in our plasma reactor when using a higher surface area quartz wool support for the catalyst bed than a fritted tube. Electrostatic interactions rely on particle chemistry, geometry, and plasma properties; while a full analysis is impossible, the review by Neyts and our recent work by Tiwari et al. support our observations.^42,43^ The charging effect can both increase and decrease the reaction rates of interest.^41^

**Figure 6:**
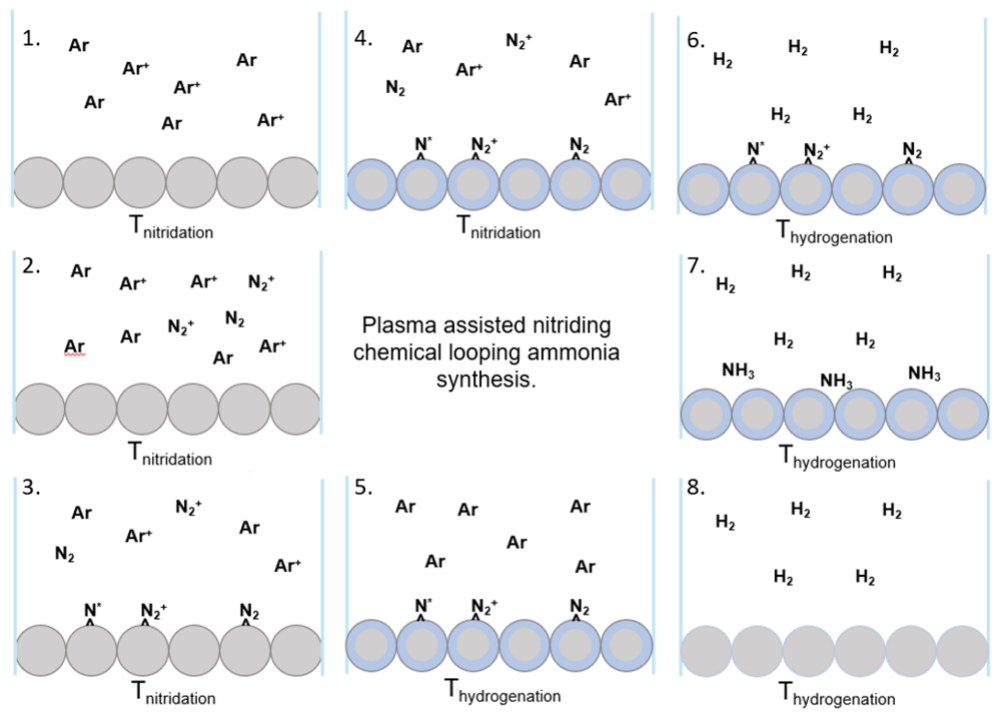
Plasma–surface mechanism. Stepwise plasma-enhanced CLAS reaction with idealized catalyst bed and gaseous species. (1) Ar plasma is initiated in the waveguide, (2) N_2_ is introduced into the plasma stream, (3) activated nitrogen interacts with the catalyst surface, (4) after the time of nitridation, a layer of metal nitride forms on the catalyst, (5) Ar is purged through the system to remove gaseous N_2_, and the temperature is changed to the temperature of ammonia synthesis, (6) H_2_ is introduced into the system, (7) H_2_ reacts readily with the nitride catalyst at the temperature of hydrogenation, and (8) once the catalyst is spent, very little nitrogen remains in the lattice.

A simplified reaction mechanism is proposed in Figure 6. The entire reaction process is visualized in the illustration, so dimensions are not accurate or to scale. In Figure 6 panel (1), the catalyst bed is brought to the temperature of nitridation (*T*_nitridation_), and the plasma is initiated. In Figure 6 panel (2), after the plasma is stable, N_2_ is introduced into the system, and the nitridation reaction begins. In Figure 6 panel (3), activated nitrogen species accumulate on and interact with the surface. In Figure 6 panel (4), a nitride diffusion layer is present; in a real system, this would be likely impacted by grain boundaries and morphology, but here, it is idealized as spherical. Once the processing time is complete, the plasma is stopped, and nitrogen is purged from the system with Ar flow, as seen in Figure 6 panel (5), surface absorbed species may remain. Next, in Figure 6 panel (6), the temperature is adjusted to the hydrogenation temperature, and H_2_ is added. In Figure 6 panel (7), ammonia is liberated from both the surface due to the ease of H_2_ dissociation and diffusion in Fe in the bulk. Finally, in Figure 6 panel (8), the reaction decreases as most of the lattice nitrogen is removed, and the experiment is ended.

The steps in plasma nitridation by N_2_ of steels and iron samples are physisorption, direct chemisorption, bulk phase dissociation route, and ion implantation.^44^ Typically, in an MWP process, this process is limited by atomic nitrogen formation, followed by the activation energy of the diffusion of the N atoms into the lattice from the surface and subsurface layers.^44^ To determine the impact of lattice diffusion at operational conditions and temperatures, thermodynamic simulations were performed via density functional theory (DFT) calculations of the N_2_/Fe system using α-phase Fe (BCC) surfaces. Upon longer deep reduction of samples nitrided at 150, 250, and 300 °C, a second “lattice”-like peak of ammonia generation is detected upon increasing temperature from 450 to 800 °C. This seems to suggest that our surface–lattice-mediated hypothesis may provide some elucidation of the dominating process occurring in plasma-mediated CLAS.

### Computational Analysis

To advance the in-depth understanding of the volcano-like ammonia productivity under plasma conditions (Figure 3), we determined the potential equilibrium constants of nitrogen (N*) species diffusion and reduction within the Fe catalysts as a function of temperature via performing DFT simulations with statistical mechanics calculations. Based on the DFT calculations, N* species on the top of the Fe surface at the examined coverages (i.e., 1/16 monolayer (ML) to 1/2 ML) were found to be the most thermodynamically favorable to be formed compared to that at the subsurface or in the bulk (Figures S11–S17). Thus, the diffusion of N* from the surface to the subsurface or the bulk is energetically unfavorable.

**Figure 7:**
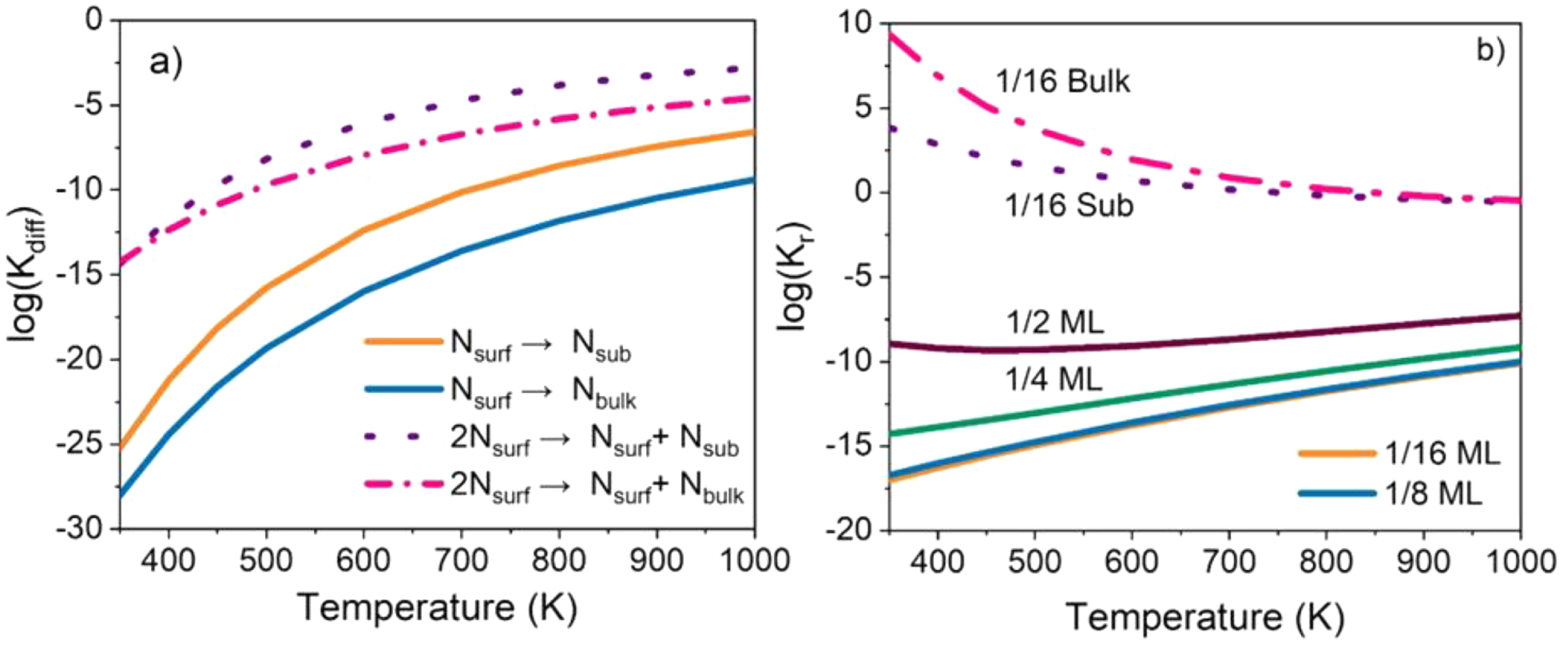
Equilibrium constants of the diffusion and reduction of N* species within the Fe(100) catalyst as a function of temperature. (a) Equilibrium constants of diffusion per N* species at 1/16 ML from the surface to the subsurface or the bulk in the presence (solid line) and absence (dotted line) of the pre-adsorbed 1/16 ML surface N* species. (b) Equilibrium constants of reduction per nitrogen (solid lines) by hydrogen to form ammonia at different surface N* coverages; N* species reduction at 1/16 ML in the subsurface (dotted line) and N* species reduction at 1/16 ML in the bulk (dot–dashed line).

We then calculated the equilibrium constants for N* species diffusion from the surface to the subsurface or bulk at different coverages as a function of temperatures, as shown in Figures 7a and (Figure S18). Our results show that as the temperature increases, N* on the surface becomes more thermodynamically favorable to diffuse to the subsurface and bulk. In addition, as the surface N* concentration increases, N* on the surface becomes more thermodynamically favorable to diffuse to the subsurface and bulk. This indicates that with increasing temperature and pressure of the N_2_, the concentration of subsurface and bulk N* will potentially increase.

In addition, we calculated the N* species reduction by hydrogen (H_2_) to form ammonia (NH_3_) at different concentrations and different locations within the Fe catalysts. Based on the DFT calculations, the potential rate-limiting step is the surface N* reduction by H_2_. As the surface coverage increases, the reduction energy per surface N* decreases due to the repulsive lateral interaction (Figure S12). Since the diffusion (Table S1) of N* from the subsurface or bulk to the surface is energetically favorable, this leads to the reduction energy per N* at the subsurface or bulk being exothermic. In the presence of subsurface or bulk N*, the reduction energetics of surface N* by hydrogen to form ammonia are relatively more favorable as compared to those without subsurface or bulk N* due to the repulsive lateral interaction (Table S2).

Furthermore, we did the statistical mechanics analysis on the equilibrium constants for the potential rate-limiting step of N* species over the Fe(100) surface reduction by H_2_ to form NH_3_ as a function of temperature (Figure 7b). Our results show that at the surface coverage (1/16 ML to 1/2 ML), since the Gibbs free energy of surface N* reduction by H_2_ to form NH_3_ is an endothermic reaction, the equilibrium constant of surface N* species reduction is much smaller than 1. As the temperature increases, the surface N* reduction equilibrium constant increases.

When the N* species locates at the subsurface or in the bulk, the equilibrium constant of surface N* species reduction increases as compared to the surface N* due to the highly exothermic diffusion energy of nitrogen from the subsurface or bulk to the top of the surface. In addition, the results show that at a lower temperature, the reduction equilibrium constants for all the examined subsurface or bulk N* species at different coverages are higher than those at a higher temperature. Taking these reduction results together, the potential rate-limiting step of the reduction process is the surface N* reduction. With the bulk or subsurface N* within the Fe catalysts, the reduction of N* to ammonia will potentially be limited by thermal equilibrium at high temperatures.

In summary, the theoretical results (Figures 7, and Figure S18) correspond well with the experiments (Figure 3) that at lower temperatures, there are less N* species in the Fe catalyst than that at higher temperatures, while at higher temperatures, the equilibrium limits the N* reduction by H_2_ to form NH_3_. More theoretical details can be found in the Supporting Information.

## Conclusions

While this reaction mechanism is generated in the context of a CLAS Fe material, it can also be generalized for other simple metal-based nitrogen carriers without specific catalytic islands or promoters. However, the affinity toward surface nitrogen may differ, as well as the temperature required to form nitride bonds. This particularly impacts nitrides of Mn and CoMo, which tend to be more thermodynamically favorable than Fe.^45^ However, this may result in longer nitridation times as more “catalytic nitrides” may have better kinetic properties.^25^ As CLAS process cycle times shorten more to match those of chemical looping combustion and process conditions become milder, this approach may be preferable. Nitrogen reactions that fall into a “surface-mediated” rather than “bulk-mediated” regime may overcome some of the energy requirements for lattice diffusion. Eventually, as the processing time is shortened, it approaches the time scale of transport limitations much sooner than those of ambient-pressure HB reactions. Thus, there likely exist some optimum conditions between the two, CLAS and ambient-pressure HB reactions.

Time-on-stream chemical looping experiments were carried out to evaluate the efficacy of pre-activation of nitrogen by MWP and to study the inherent kinetics of nitrogen storage materials by solely thermochemical means. Nitrogen plasma is found to enhance the overall reaction productivity and reduce temperatures of the nitridation reaction by first pre-activating nitrogen before depositing it on the surface of the catalyst. Rates of up to 420.9 μmol min^–1^ g^–1^ at 250 °C nitridation temperature were found to be optimal. Additionally, a surface-mediated and bulk-mediated reaction domain was found to exist depending on the length of the plasma-treatment times. The existence of this feature can be related to surface nitrogen accumulation, particle morphology, catalyst bed temperature. This is partially validated by the associated DFT calculations. At a higher temperature, the nitrogen species are easier to diffuse to the sublayer or bulk of the iron catalysts than the lower temperature case. While the higher temperature limited the equlibrium constant of the nitrogen speices in the sublayer or bulk of the iron catalyst reduction to form ammonia than the lower temperature scenario. One of the features, which has been lacking for the past 10 years of low-pressure CLAS, is the absence of well-established thermochemical fixed-bed kinetics. Much material development work has been performed but with little study beyond fixed rates.

The aim of this study is to evaluate the effect of plasma on the system and to benchmark the traditional catalytic materials for nitrogen fixation by chemical looping for effective comparisons between catalysts to be drawn. There still exist many open questions in CLAS reaction engineering, such as materials design, particle attrition, reactor design, and modeling questions of kinetics, cycle times, and techno-economic analysis. As well as the development and application of more advanced models of gas–solid reactions developed for CLC catalysts, these approaches may be borrowed from the more advanced combustion field, although detailed reaction data are still not fully developed. This work has aimed to begin answering some of those critical questions on kinetics with both an MWP system and the traditional thermochemical fixed-bed approach. We have shown that plasma pre-treatment results in better ammonia productivity, lower processing temperatures, shorter reaction times, and higher rates.

## Supporting Information Available

Diagram of the reactor apparatus, reaction engineering equations, power-law kinetics, SCM, activation energy, OES, XRD, SEM, main plasma reactions, and further details on computation methods (PDF)

## Abbreviations

CLAS: chemical looping ammonia synthesis
DFT: density functional theory
EDX: energy-dispersive X-ray spectroscopy
FWHM: full width at half max
HB: Haber–Bosch reaction
hyd: hydrogenation
ML: monolayer
MWP: microwave plasma
nit: nitridation
OES: optical emission spectroscopy
red: reduced
SCM: shrinking core model
SEM: scanning electron microscopy
XRD: X-ray diffractometry
