# Supplementary material for: Ambient Carbon-Neutral Ammonia Generation via a Cyclic Microwave Plasma Process
Source: ACS Appl Mater Interfaces. 2023 May 3;15(19):23255–64. doi: 10.1021/acsami.3c02508 (PMC10197069; doi:10.1021/acsami.3c02508)
Supplement: Supplementary file 1 — am3c02508_si_001.pdf [file am3c02508_si_001.pdf]

Supporting Information for:

Ambient carbon-neutral ammonia generation via a cyclic microwave plasma process

Sean Brown[1], Saleh Ahmat Ibrahim[2], Brandon R. Robinson[1], Ashley Caiola[1], Sarojini Tiwari[1], Yuxin Wang[1], Debangsu Bhattacharyya[1], Fanglin Che[2]*, Jianli Hu[1]*

[1]Department of Chemical and Biomedical Engineering, Benjamin M. Statler College of Engineering and Mineral Resources, West Virginia University, 395 Evansdale Drive, Morgantown, WV 26505, USA

[2]Department of Chemical Engineering, Francis College of Engineering, University of Massachusetts Lowell, One University Avenue, Lowell, MA 01854, USA

*Contact:
John.Hu@mail.wvu.edu
Fanglin_Che@uml.edu

Reactor Schematic

The microwave plasma reactor schematic is presented in Figure S1. The plasma reactor apparatus, the plasma jet above but not in contact with the catalyst bed, the OES at the surfaguide outlet choke, and tubular furnace surrounding the catalyst bed.

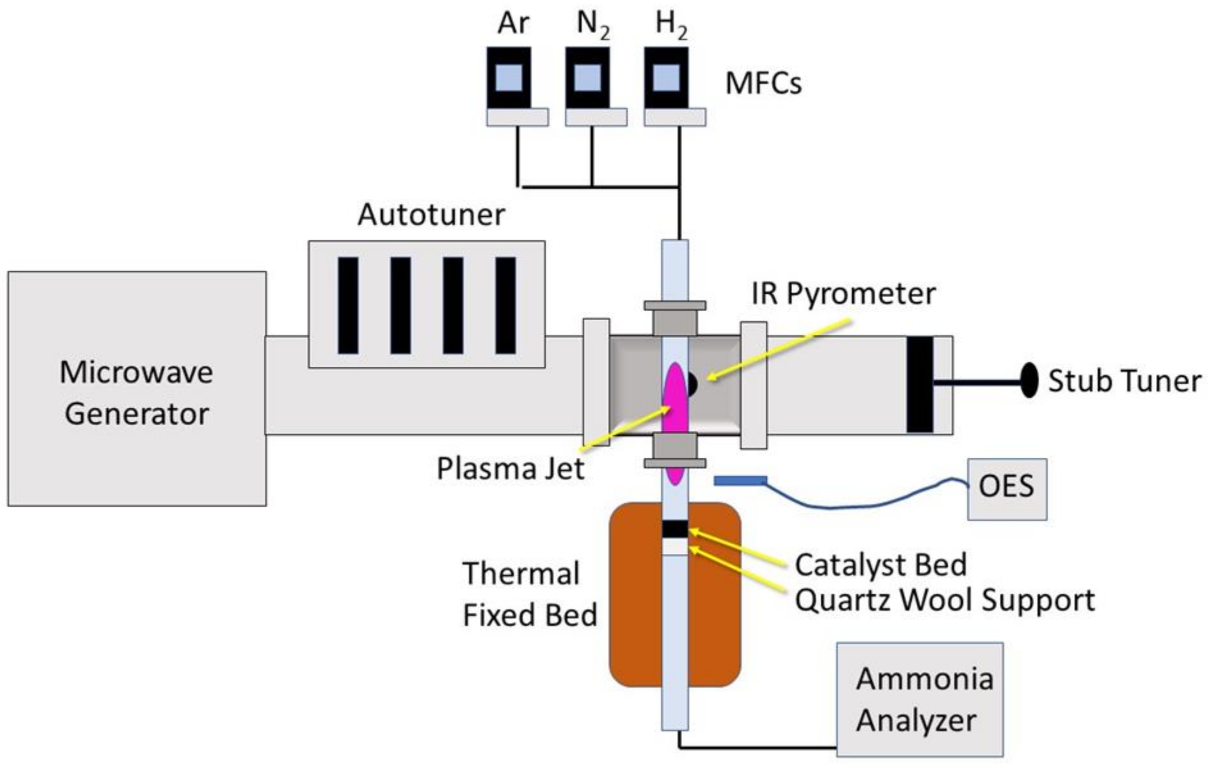

Figure S1. The plasma reactor.

Materials Characterization

X-ray diffraction patterns were collected for the fresh Fe and plasma-nitrided Fe samples after 1 h time on stream at 250 C, 10 sccm N_2, 40 sccm Ar, at 0.3 kW (Figure S9.). Little bulk change is observed from the XRD pattern, lending evidence to the surface and near surface reaction mechanisms.

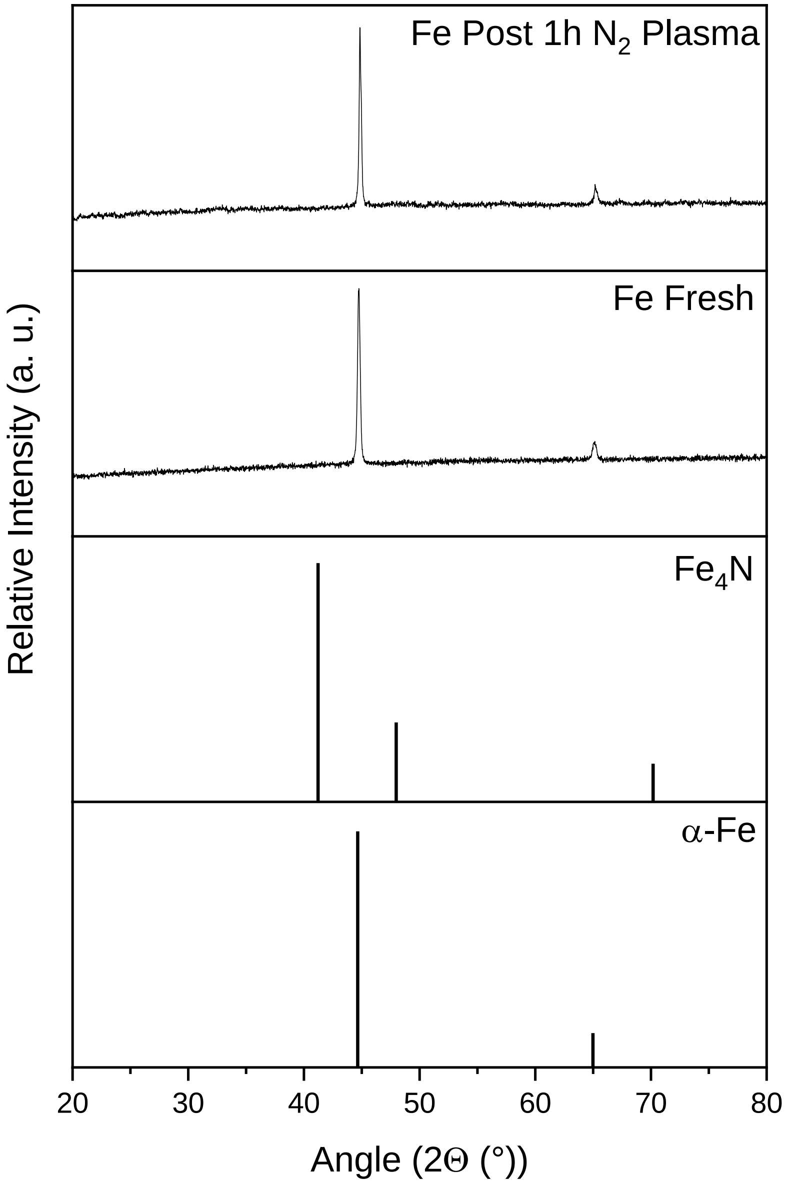

Figure S2. X-ray diffraction patterns for both fresh Fe powder and plasma nitrided samples.

Scanning electron microscopy was used to investigate particle morphology, very little evidence of corrosion or sintering was discovered. EDX was also used to evaluate the nitrogen content in the nitrided samples.

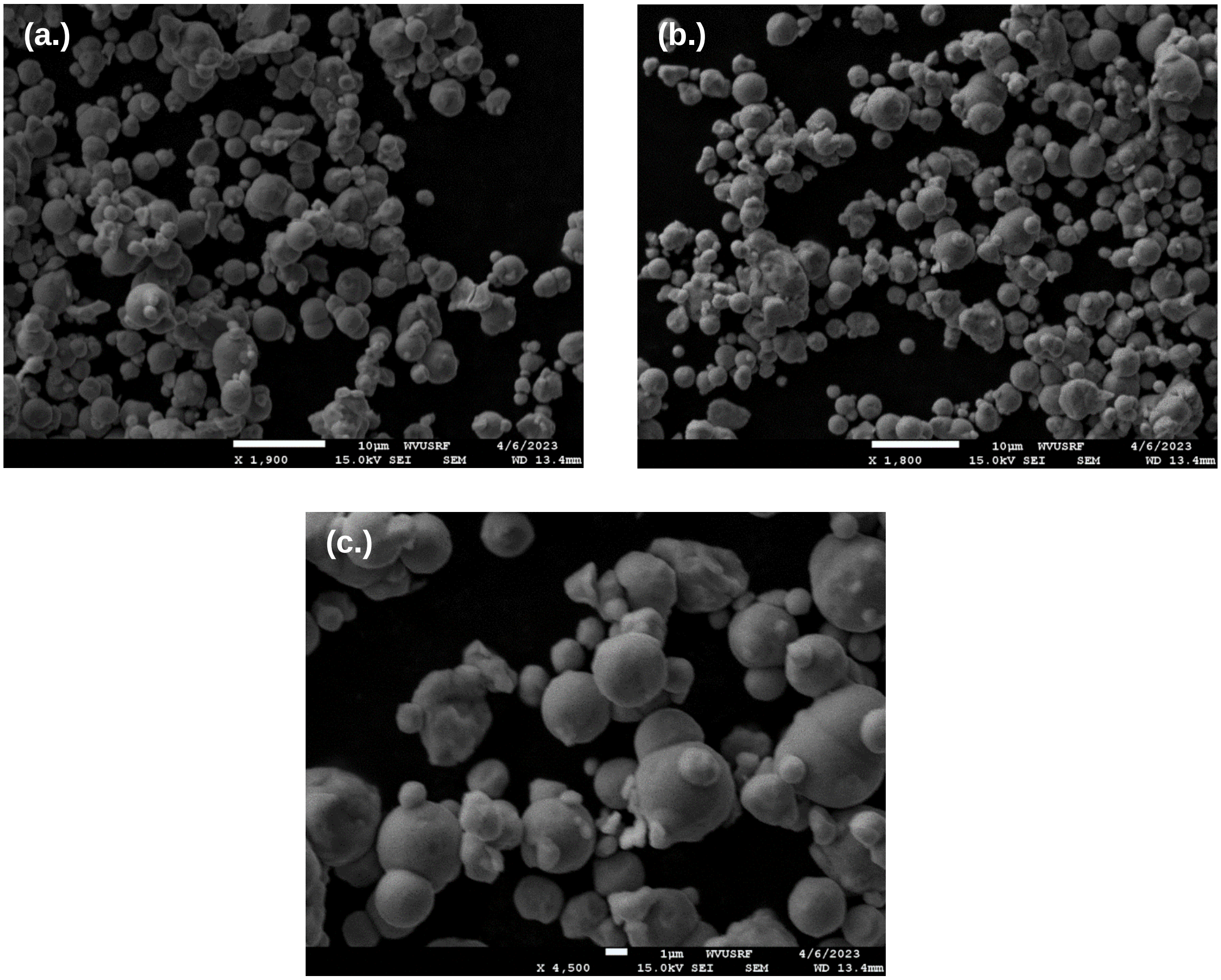

Figure S3. Electron microscopy of the Fe particle morphology after plasma nitridation.

The nitrided particles were also examined using elemental mapping with EDX, the results indicated minimal nitrogen content, with the majority products as C and O. This result follows our experimental observation of the surface and sub-surface nitrogen species as being very reactive.

Plasma Iron Reaction Engineering

In lieu of estimating the nitrogen content of each treated sample with TGA or XRD and risking exposure to air, the concentration of ammonia synthesized over a given reaction time was integrated as (C_{total}), Equation S2. In this way conversion (X), Equation S1, could be determined and plotted as a function of time by the simple ratio of the C(t) in ppm over the integrated concertation.

$$X = \frac{C(t)}{C_{total}} \tag{S1}$$

$$C_{total} = \int_{t_0}^{t_f} C(t)dt \tag{S2}$$

$$r = kP_{H_2}^{n} \tag{S3}$$

$$\ln(r) = \ln(k) + nln(P_{H_2}) \tag{S4}$$

Similarly, reaction rates were calculated by converting the concentration in ppm to μmoles and dividing by time of reaction and normalizing by mass of catalyst.

Apparent activation energies, Figure S2, were calculated by using the Arrhenius plot method. Kinetic parameters were calculated using the power law kinetic model with Equation S3 and Equation S4. A plot of a best fit line using the power law model for the plasma enhanced catalytic system are presented in Figure S3.

The power law model determined that order is n = 0.72 (approaching first order), and k = 12.92 s^{-1} with an R^2 of 0.9187.

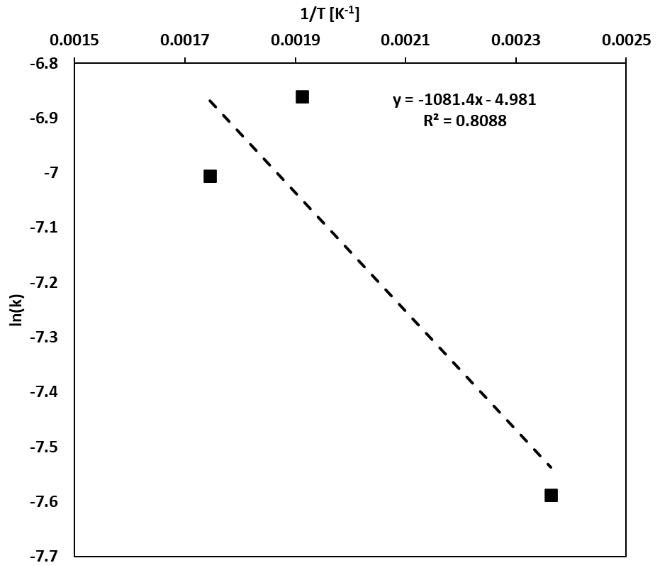

Figure S4. Plasma nitrided activation energy plot. Apparent activation energy of ammonia synthesis over plasma nitrided Fe by the Arrhenius plot method.

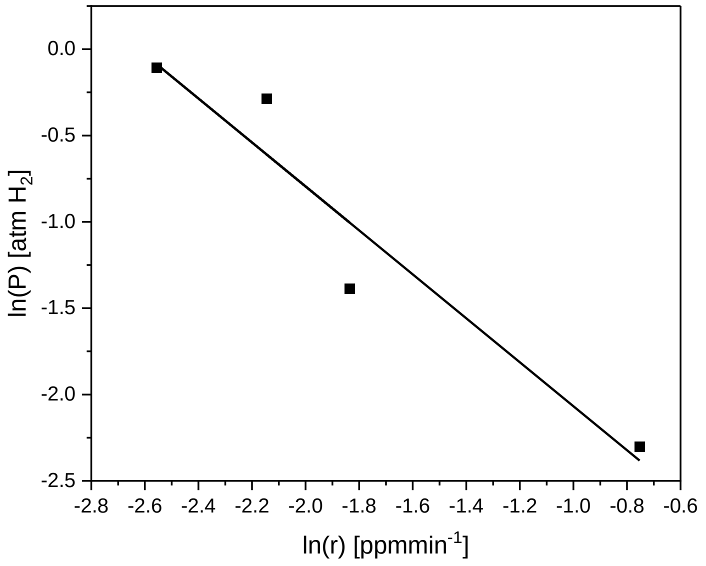

Figure S5. Plasma kinetics by the power law. Representative kinetics of the ammonia synthesis process over plasma nitrided Fe particles.

Thermal Fixed Bed Reaction Engineering

Shrinking core model equations were used to analyze the thermal fixed bed kinetics of Mn, Fe, and CoMo as chemical looping ammonia synthesis materials.[1,2] These equations fit three different rate determining steps in a gas-solid reaction of unchanging volume, Equation S5, the gas diffusion limit, Equation S6, the bulk diffusion limit, and Equation S7, the surface reaction limit. Where t is the time in s, k is the rate constant in s^{-1}, and X is the conversion.
The results presented for the Arrenhius model and SCM fits are all based on data collected from fixed bed experiment runs (n = 3 or n = 5) times.

$$tk_{gas} = X_{NH_3} \quad \text{(S5)}$$

$$tk_{bulk} = 1 - 3\left(1 - X_{NH_3}\right)^{\frac{2}{3}} + 2(1 - X_{NH_3}) \quad \text{(S6)}$$

$$tk_{surface} = 1 - (1 - X_{NH_3})^{\frac{1}{3}} \quad \text{(S7)}$$

Representative plots of these reactions with best fit lines are presented in Figure S4 for each sample and condition evaluate.

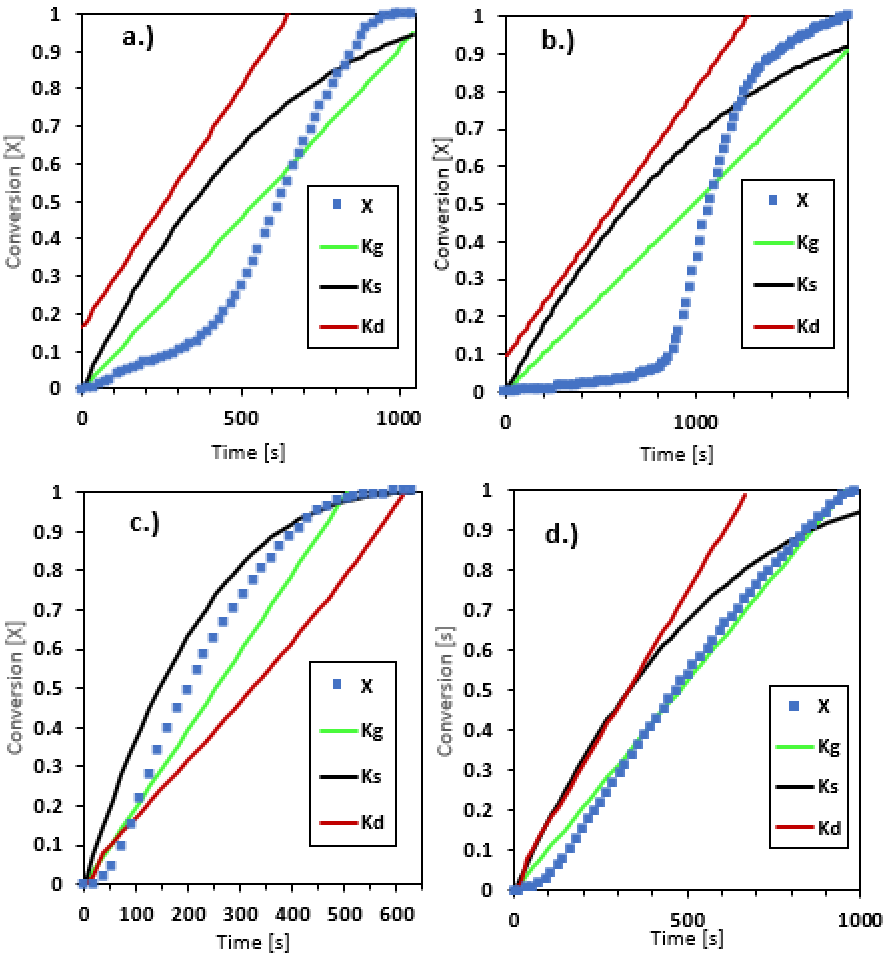

Figure S6. Shrinking core plasma kinetics. Representative kinetics of the ammonia synthesis process over thermally nitrided Fe particles, a.) 300 °C, b.) 250 °C, c.) 200 °C, and d.) 150 °C.

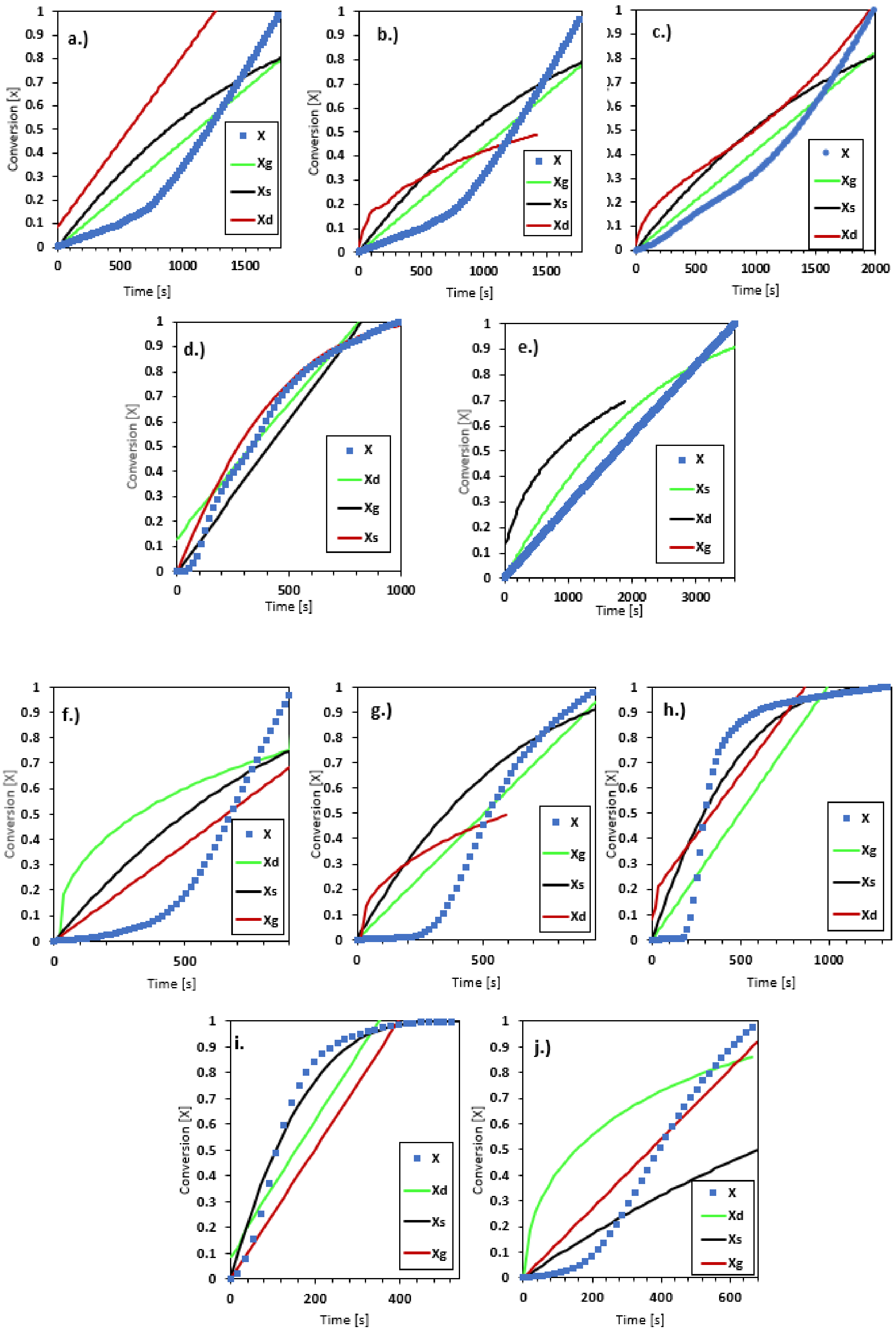
a.)
Conversion [X]
Time [s]
X
Xg
Xs
Xd
b.)
Conversion [X]
Time [s]
X
Xg
Xs
Xd
c.)
Time [s]
X
Xg
Xs
Xd
d.)
Conversion [X]
Time [s]
X
Xd
Xg
Xs
e.)
Conversion [X]
Time [s]
X
Xs
Xd
Xg
f.)
Conversion [X]
Time [s]
X
Xd
Xs
Xg
g.)
Conversion [X]
Time [s]
X
Xg
Xs
Xd
h.)
Conversion [X]
Time [s]
X
Xg
Xs
Xd
i.
Conversion [X]
Time [s]
X
Xd
Xs
Xg
j.)
Conversion [X]
Time [s]
X
Xd
Xs
Xg

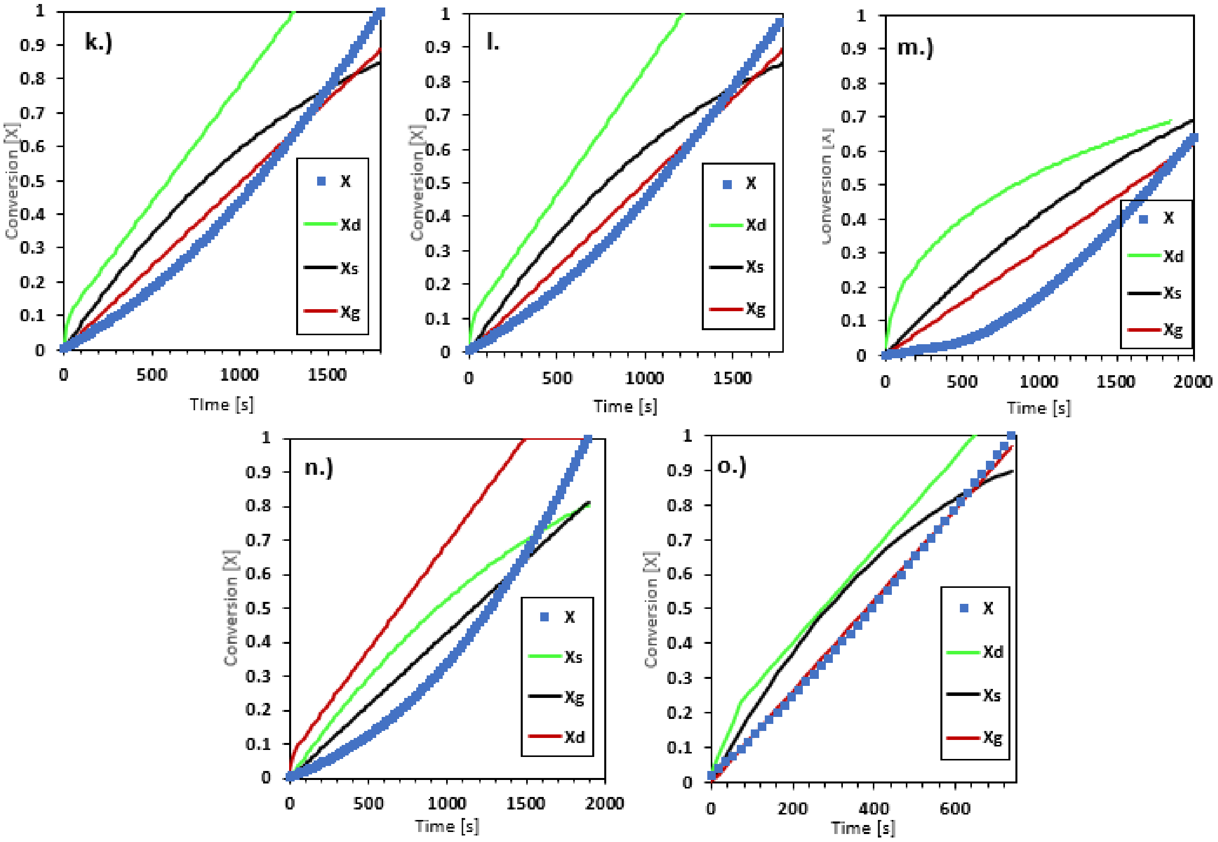

Figure S7. Shrinking core thermal kinetics. Representative kinetics of the ammonia synthesis process over thermally nitrided particles. CoMo a.) 450 °C 25 sccm, b.) 450 °C 100 sccm, c.) 550 °C, d.) 450 °C, e.) 350 °C. Fe, f.) 250 °C, g.) 350 °C, h.) 400 °C, i.) 450 °C, j.) 500 °C. Mn k.) 300 °C, l.) 350 °C, m.) 450 °C, n.) 500 °C, o.) 450 °C 100 sccm.

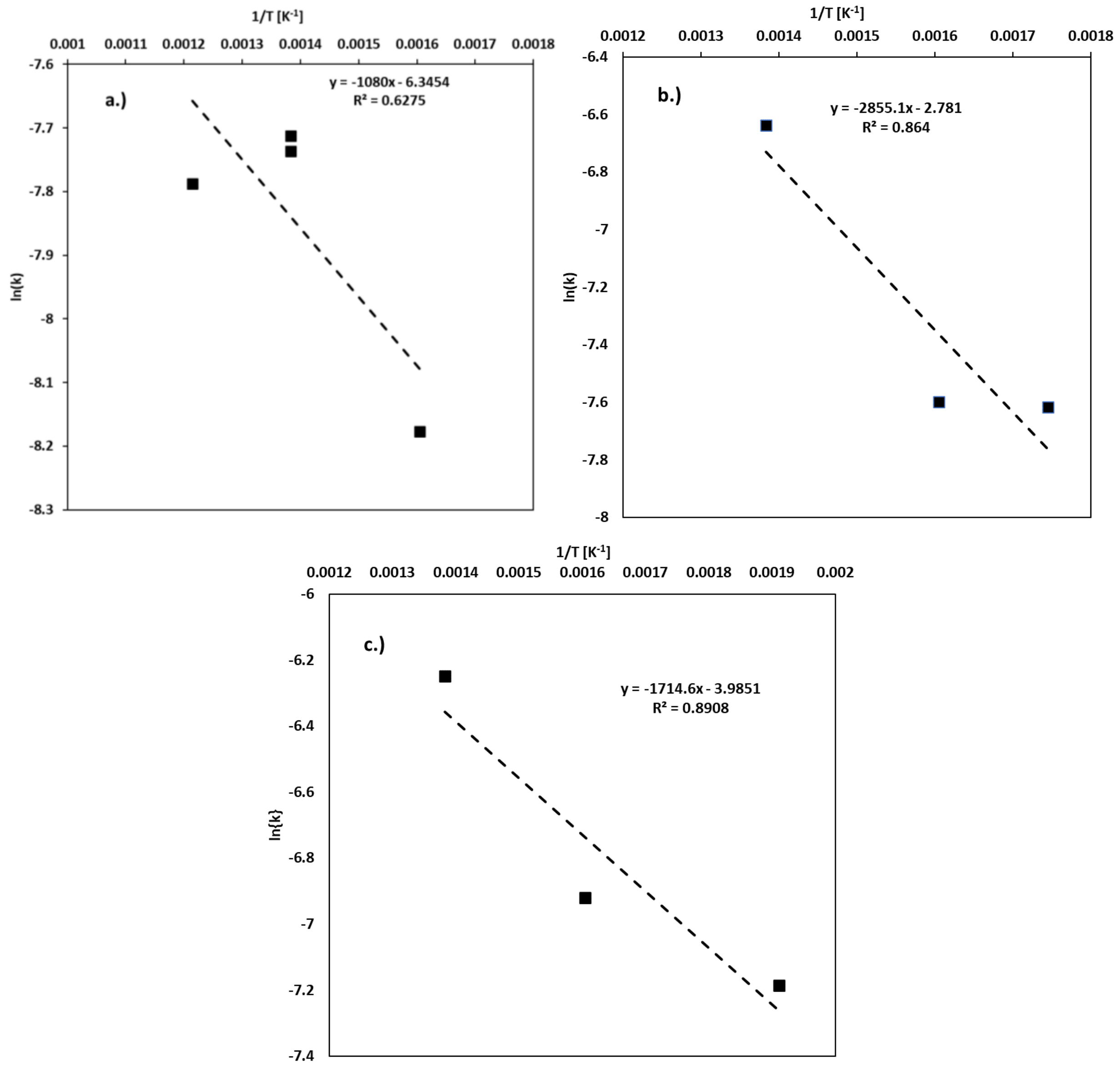

Figure S8. Shrinking core model activation energy. Apparent activation energies were calculated by using the Arrhenius plot method for each catalyst treated thermally, a.) CoMo, b.) Mn, c.) Fe.

Gas-solid reactions of this type typically have more complex activation energy relationships. Prior literature has not yet moved beyond the SCM and Arrenhius plot. In chemical looping ammonia synthesis under plasma, we are the first who collected high resolution time-on-stream data (blue dotted line in Figures S4). Also, this is first reporting of using SCM to model plasma-assisted ammonia chemical looing synthesis. the main focus of this paper is not a theoretic one. Our future modeling focused work can address this by applying oxygen carrier models to nitrogen chemistry.

Plasma Characterization

Optical emission spectra were collected and evaluated to characterization the plasma temperature and the number of activated nitrogen species.

Electron temperature of plasma can be determined by the Boltzmann plot method, Equation S8.[3] This is performed for both sets of spectra, Ar and Ar:N_2, Figures S7 and S8. The approximate number of N_2^+ in low temperature, Equation S9, plasma can be directly related to the intensity observed of the transition at ~391 nm, Equation S10.[4]

$$\ln\left(\frac{I_{ki}\lambda_{ki}}{g_k A_{ki}}\right) = -\frac{E_k}{k_B T_e} + C \qquad \text{(S7)}$$

$$I\,(N_2^+, \lambda = 391.4\,nm) \approx N_u^+ \qquad \text{(S8)}$$

$$N_2^+(B^2\Sigma_u^+) \rightarrow N_2^+\left(X^2\Sigma_g^+\right) + h\upsilon \qquad \text{(S9)}$$

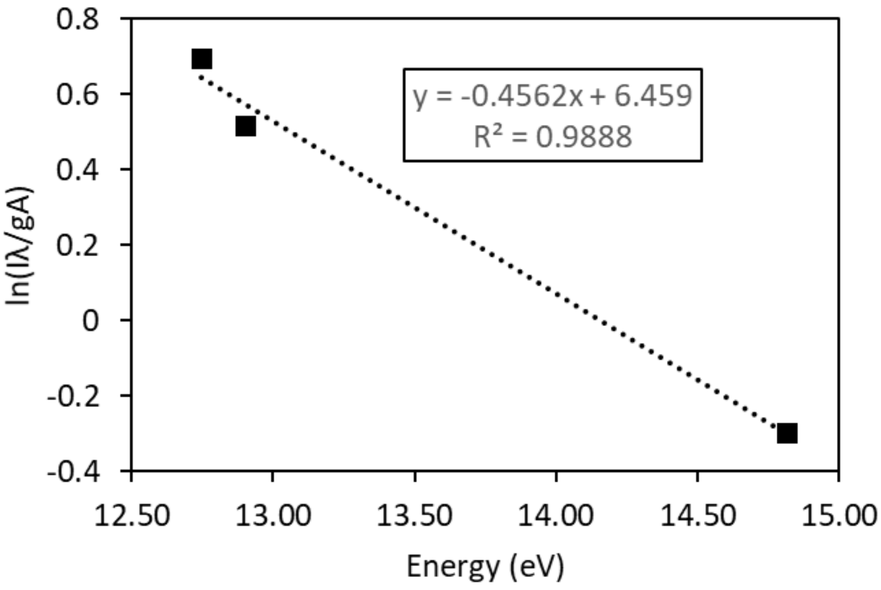

Figure S9. Electron temperature. The Boltzmann plot for the Ar I spectrum obtained in the 80%, 20% Ar:N_2 plasma.

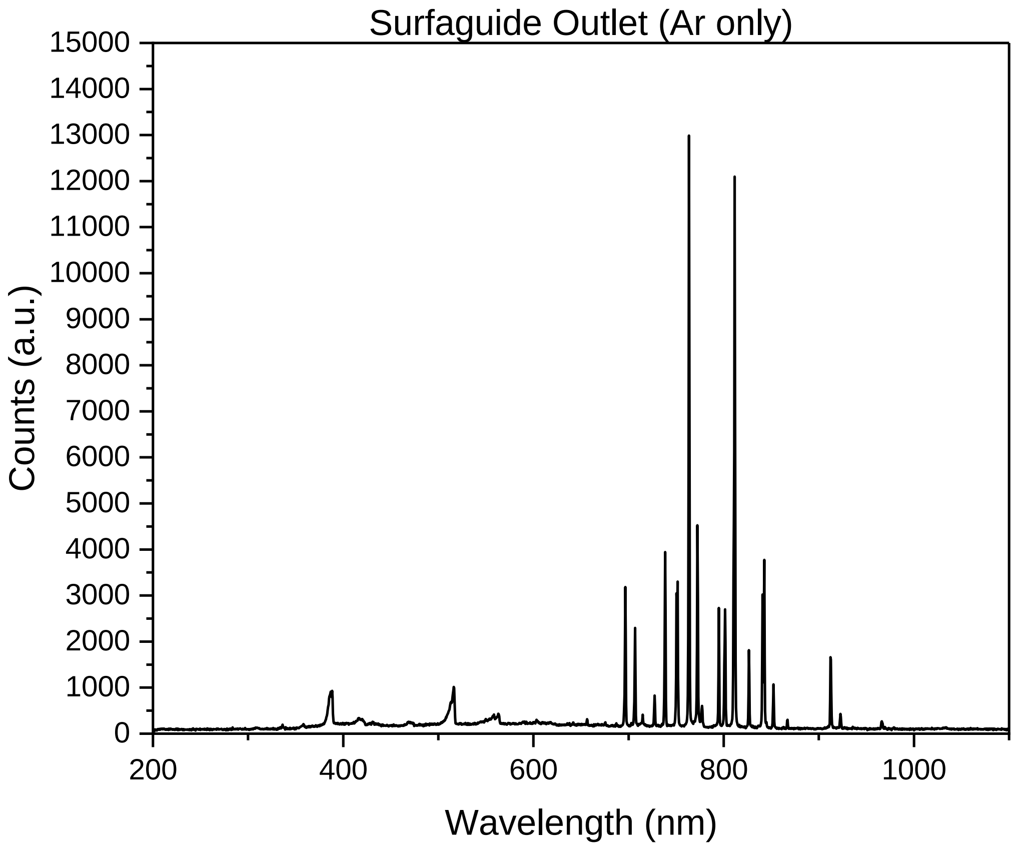

Figure S10. Spectra collected from center of the waveguide port.

Proposed reactions to form the observed transition at ~391 nm in Equation S8. In Equation S11 the direct conversion from neutral N_2 to the activated N_2^+ species is observed.[4] The more likely route in a low temperature plasma is the combined reaction Equation S12 followed by Equation S13.[4] The energy of the final transition step, Equation S13, is only 3.13 eV.[4]

$$N_2\left(X^1\Sigma_g^+\right) + e^-(E > 18.7\ eV) \rightarrow\ N_2^+(B^2\Sigma_u^+) + 2e^- \quad \text{(S11)}$$

$$N_2\left(X^1\Sigma_g^+\right) + e^-(E > 15.57\ eV) \rightarrow\ N_2^+(X^2\Sigma_u^+) + 2e^- \quad \text{(S12)}$$

$$N_2^+\left(X^2\Sigma_g^+\right) + e^-(E > 3.13\ eV) \rightarrow\ N_2^+(B^2\Sigma_u^+) + e^- \quad \text{(S13)}$$

Atomic nitrogen emissions, N^* ~425 nm, is difficult to distinguish from the Ar I excited state ~ 419.8 nm in the collected spectra. The energy of each transition is very similar; however, the lifetime of these species is very short, 39 ns for Ar [4].

Average velocity in the reaction tube was determined by the flow rate, Q,50 sccm, and the cross-sectional area, A, of the tube using Equation S14. The average lifetime of the N_2^+ species, 67 ns, was compared with the length between the plasma and the catalyst, z = 3 cm, and the average fluid velocity, v, 1.5 s. Additional activated states of the N_2^+ molecule which can arise from collisions have life spans between 10, 1, 0.7 s, and 0.17 ms.[5]

$$Q = vA \tag{S14}$$

Computational Method

Using the Vienna Ab-initio Simulation Package (VASP), [6,7] density functional theory (DFT) calculations were carried out. These calculations were used to determine the electronic energy and the configuration with the most favourable adsorption configurations for nitrogen (N) species within an iron (Fe) catalyst. To describe the electron exchange-correlation and the ion-electron interactions, the Perdew-Burke-Ernzerhof (PBE)[8] functionals and the projector-augmented wave (PAW) [9,10] methods were used. The Monkhorst-Pack mesh of (3 × 3 × 1) k-points and the plane-wave energy cutoff of 500 eV were applied to a four-layer p(4 × 4) Fe surface. To facilitate electronic interaction with the adsorbates, the top two layers of the catalytic surface were allowed to relax while the bottom two remained in their bulk positions. When the electronic energy converged to 10^{-5} eV and the ionic forces were less than 0.03 eV/Å, the configurations were optimized. Spin polarization has been counted for all the calculations. The calculated lattice constant of Fe is 2.86 Å , which is similar to the experimental report[11] of 2.858 Å and theoretical report[12] of 2.85 Å. The most stable Fe facet, Fe(100), was used to perform the DFT calculations. N^* species can adsorb at one of three sites[13] on the Fe(100) surface, including the top, bridge, and hollow sites (Figure S11).

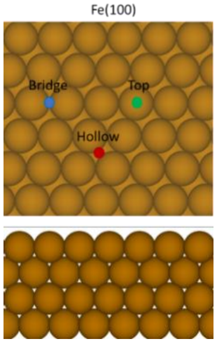

Figure S11. Top and side views of the Fe(100) catalytic surface with various possible adsorption sites, including top, bridge, and hollow sites.

Based on the adsorption calculations (Eqn. (S15)),[14] the most favorable adsorption site for N^* over Fe(100) is the hollow site, with the adsorption energy of -1.6 eV.

$$E_{ad} = E_{total} - E_{surface} - E_{adsorbate} \quad \text{(S15)}$$

here E_{total} corresponds to the total energy of the species over the surface, $E_{surface}$ represents the energy of the surface and $E_{adsorbate}$ represents the energy of the adsorbate in the gas phase.

DFT calculations were employed to determine the formation ($\frac{n}{2}N_2(g) + n* \rightarrow nN^*$) and reduction ($nN^* + \frac{3n}{2}H_2(g) \rightarrow nNH_3(g) + n*$) energies of nitrogen species at different concentrations (represented by coverages in the unit of monolayer (ML) and at different locations namely, the surface, the subsurface and the bulk. The energies of formation per N^* (ΔE_f) and the reduction per N^* (ΔE_r) within Fe(100) are calculated using Eqns. (S16) - (S17).

$$\Delta E_f = \frac{E(nN^*) - E(*) - \frac{n}{2}E(N_2)}{n} \quad \text{(S16)}$$

$$\Delta E_r = \frac{nE(NH_3) + E(*) - E(nN^*) - \frac{3n}{2}E(H_2)}{n} \quad \text{(S17)}$$

where $E(nN^*)$ represents the total energy of n number of N^* species in an Fe(100) catalyst, $E(*)$ represents the energy of the Fe(100) surface, and $E(N_2)$, $E(NH_3)$, $E(H_2)$ represent the gas phase energies of N_2, NH_3 and H_2 molecules, respectively.

The energies of formation per N^* and reduction per N^* in an Fe(100) catalyst at different coverages and at different locations are presented in Figure S12.

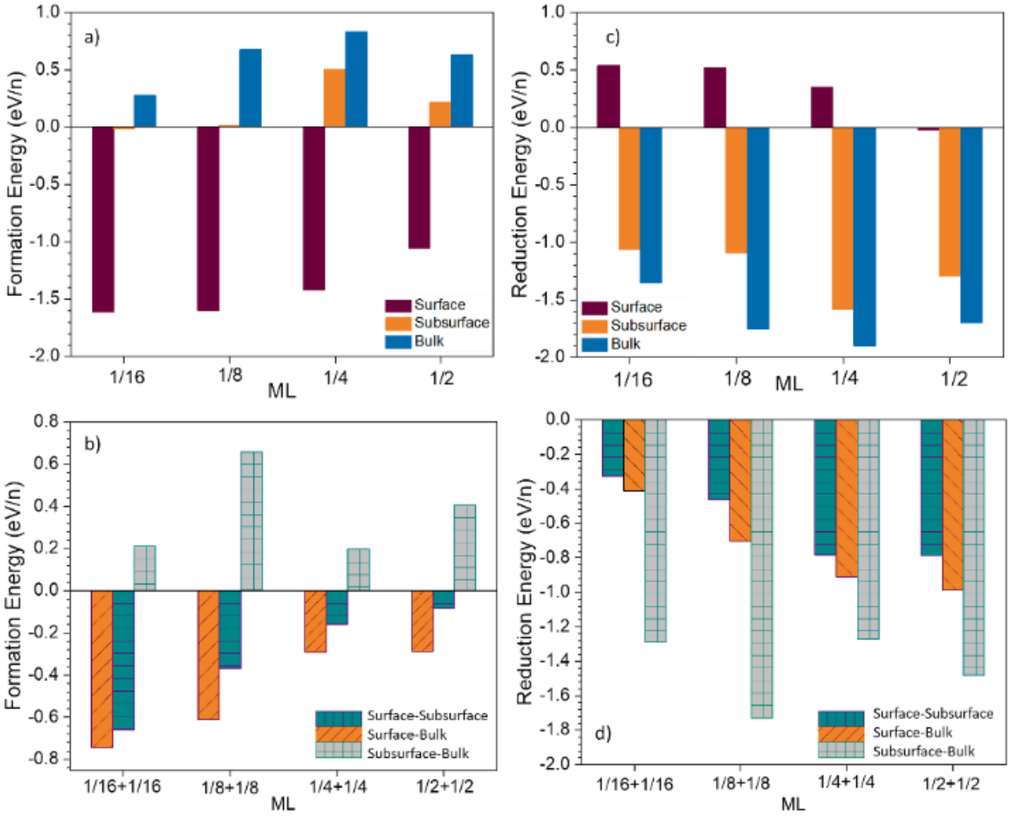

Figure S12. The formation energy per N* and reduction energy per N* at various coverages and different locations within the Fe(100) catalytic surface.

Figure S12a-b shows that the formation energy of surface N* over Fe(100) is energetically favorable. As the surface coverage increases, the formation energy per surface N* increases due to the repulsive lateral interaction and lack of empty sites. While the diffusion of N* from the surface to the subsurface and bulk is energetically unfavorable, with the endothermic energetics in the range of 0.09 eV to 1.90 eV per nitrogen depending on the coverages and locations (Table S1). Especially, when the N* species initially adsorbed over the surface, their diffusion from the surface to the subsurface is more favorable than diffusion to the bulk. In the presence of surface (or subsurface) N*, the diffusion of N* from the surface to the subsurface or to the bulk (or from the subsurface to bulk) is relatively more favorable as compared to the one in the absence of surface (or subsurface) N* due to the repulsive lateral interaction.

The DFT-calculated electronic diffusion energy (ΔE_{diff}) of N^* species from the surface to the subsurface or to the bulk is calculated using Eqn. (S4) and is summarized in Table S1.

$$\Delta E_{diff} = \left(E_f\right)_{FS} - \left(E_f\right)_{IS} \quad \text{(S18)}$$

here $\left(E_f\right)_{IS}$ represents the initial formation energy of N^* species at the surface of the catalyst and $\left(E_f\right)_{FS}$ is associated to the formation energy of N^* species at the final state which corresponds to the formation energy of N^* species in the subsurface or in the bulk. If there are n number of N^* diffusion, we will average the diffusion energy by n.

Table S1. Diffusion energy (ΔE_{diff}) of N^* species from the surface to the subsurface or bulk at different coverages

Diffusion	ΔE_{diff} (eV) per nitrogen
$1N_{surface} \rightarrow 1N_{subsurface}$	1.60
$1N_{surface} \rightarrow 1N_{bulk}$	1.90
$2N_{surface} \rightarrow 1N_{surface} + 1N_{subsurface}$	0.42
$2N_{surface} \rightarrow 1N_{surface} + 1N_{bulk}$	0.46
$2N_{surface} \rightarrow 2N_{subsurface}$	0.80
$2N_{surface} \rightarrow 2N_{bulk}$	1.13
$4N_{surface} \rightarrow 2N_{surface} + 2N_{subsurface}$	0.20
$4N_{surface} \rightarrow 2N_{surface} + 2N_{bulk}$	0.26
$4N_{surface} \rightarrow 4N_{subsurface}$	0.48
$4N_{surface} \rightarrow 4N_{bulk}$	0.56
$8N_{surface} \rightarrow 4N_{surface} + 4N_{subsurface}$	0.09
$8N_{surface} \rightarrow 4N_{surface} + 4N_{bulk}$	0.11

Figure S12c-d shows that the reduction energy per N^* over the Fe(100) surface at different coverages and different locations. The potential rate limiting step is the surface N^* reduction by H_2. As the surface coverage increases, the reduction energy per surface N^* decreases due to the repulsive lateral interaction. Since the diffusion of N^* at the subsurface or bulk to the surface is energetically very favorable, this leads to the reduction energy per N^* at the subsurface or bulk

being < 0 eV. In the presence of subsurface or bulk N*, the reduction energetics of surface N* by hydrogen to form ammonia is relatively more favorable as compared to the one in the absence of subsurface or bulk N* due to the repulsive lateral interaction. The DFT-calculated electronic reduction energies (ΔE_r, calculated by Eqn. (S17)) per N* species at different locations and at different coverages are summarized in Table S2.

Table S2. Reduction energy (ΔE_r) per surface N* species at different coverages.

Reduction reactions	ΔE_r (eV) per N_{surf}^*
$N_{surf} + 1.5H_2(g) \rightarrow NH_3(g) + *$	0.54
$2N_{surf} + 3H_2(g) \rightarrow 2NH_3(g) + *$	0.52
$4N_{surf} + 6H_2(g) \rightarrow 4NH_3(g) + *$	0.35
$N_{surf}\_N_{sub} + 1.5H_2(g) \rightarrow NH_3(g) + N_{sub}$	0.41
$N_{surf}\_N_{bulk} + 1.5H_2(g) \rightarrow NH_3(g) + N_{bulk}$	0.52
$2N_{surf}\_2N_{sub} + 3H_2(g) \rightarrow 2NH_3(g) + 2N_{sub}$	0.17
$2N_{surf}\_2N_{bulk} + 3H_2(g) \rightarrow 2NH_3(g) + 2N_{bulk}$	0.34
$4N_{surf}\_4N_{sub} + 6H_2(g) \rightarrow 4NH_3(g) + 4N_{sub}$	0.01
$4N_{surf}\_4N_{bulk} + 6H_2(g) \rightarrow 4NH_3(g) + 4N_{bulk}$	0.08

Moreover, the adsorption configurations of N* at different coverages and different locations within the Fe(100) catalysts are shown in Figures S13-S17.

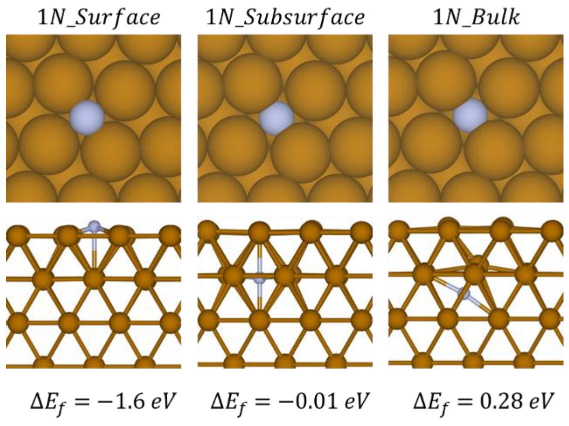

Figure S13. Top and side views of N* species at the coverage of 1/16 ML and at the different locations within the Fe(100) catalyst, including its formation at the hollow site on the surface, subsurface, and bulk layers, as well as the associated formation energies. The top and bridge sites are found to be unstable for N* species to adsorb and N* species will shift to the more stable hollow site. N* species formation at 1/16 ML is energetically more favorable on the catalyst surface than that in the subsurface and bulk layers. This suggests that the formation of N* species at 1/16 ML is not favorable in the subsurface and bulk layers and they prefer to diffuse to the surface upon adsorption.

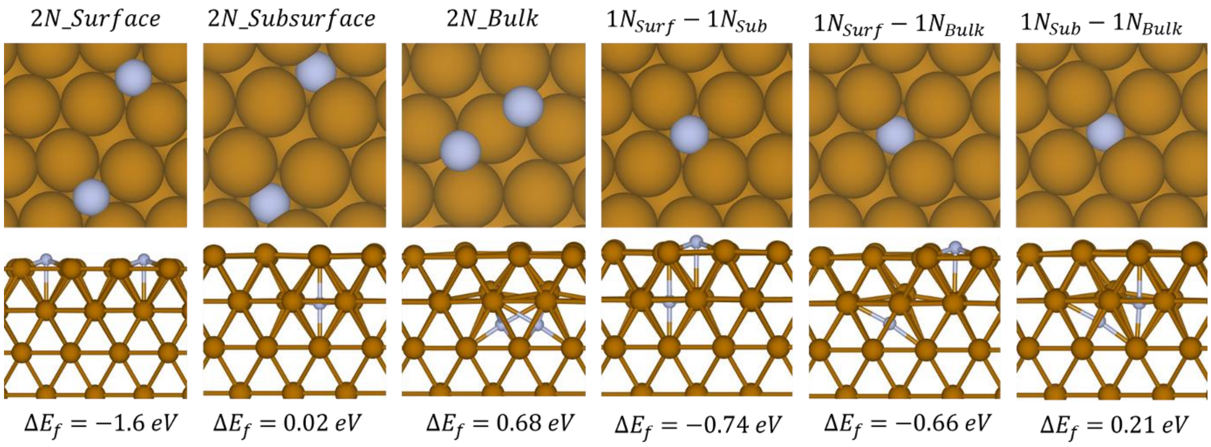

Figure S14. Top and side views of N* species at the coverage of 1/8 ML and at different locations with the Fe(100) catalyst, including its formation on the surface, subsurface, and bulk layers, as well as the associated formation energies. The formation of N* species at 1/8 ML is more energetically favorable to adsorb on the catalyst surface than that in the subsurface and bulk layers. This suggests that the formation of N* species at 1/8 ML is not favorable in the subsurface and bulk layers and they prefer to diffuse to the surface upon adsorption.

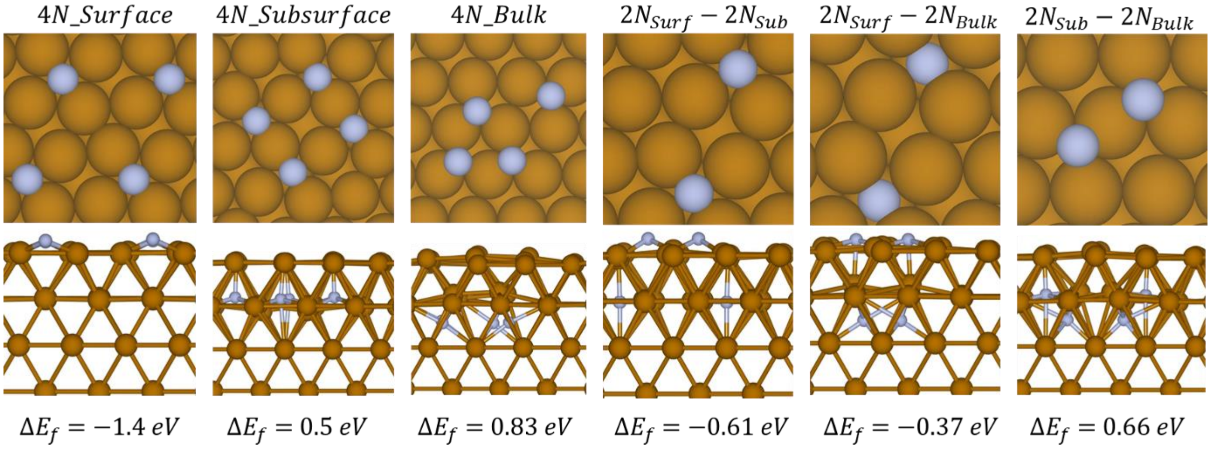

Figure S15. Top and side views of N* species at the coverage of 1/4 ML and at different locations with the Fe(100) catalyst, including its formation on the surface, subsurface, and bulk layers, as well as the associated formation energies. The formation of N* species at 1/4 ML is more energetically favorable to adsorb on the catalyst surface than that in the subsurface and bulk layers. This suggests that the formation of N* species at 1/4 ML is not favorable in the subsurface and bulk layers and they prefer to diffuse to the surface upon adsorption.

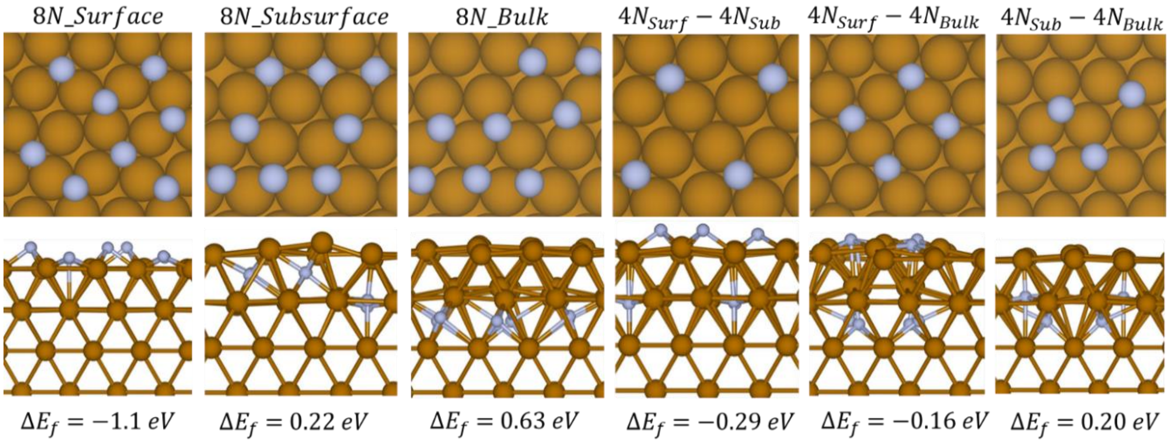

Figure S16. Top and side views of N* species at the coverage of 1/2 ML and at different locations with the Fe(100) catalyst, including its formation on the surface, subsurface, and bulk layers, as well as the associated formation energies. The formation of N* species at 1/2 ML is more energetically favorable to adsorb on the catalyst surface than that in the subsurface and bulk layers.

This suggests that the formation of N* species at 1/2 ML is not favorable in the subsurface and bulk layers and they prefer to diffuse to the surface upon adsorption.

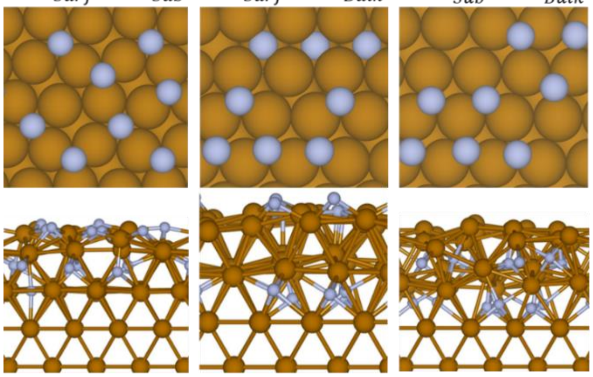

Figure S17. Top and side views of N* species at the coverage of 1/2 ML + 1/2 ML and at different locations with the Fe(100) catalyst, including its formation on the surface, subsurface, and bulk layers, as well as the associated formation energies. The formation of N* species at 1/2 ML + 1/2 ML is more energetically favorable to adsorb on the catalyst surface than that in the subsurface and bulk layers. This suggests that the formation of N* species at 1/2 ML + 1/2 ML is not favorable in the subsurface and bulk layers and they prefer to diffuse to the surface.

The calculated DFT energies were corrected to the Gibbs free energy per nitrogen of reduction (ΔG_r) and diffusion (ΔG_{diff}). The correction includes zero-point energy (ZPE), vibrational entropy (S_{vib}) and temperature (T) corrections. Here we assume that N* vibrational frequency on the surface (or the subsurface or the bulk) at different surface coverages will be the same as the one with 1/16 ML surface (or subsurface or bulk) N*, as shown in Table S3. The Gibbs free energies (ΔG_r and ΔG_{diff}) are calculated using Eqns. (S19) - (S20), respectively.

$$\Delta G_r = \Delta E_r + E_{ZPE} - TS_{vib} \quad \text{(S19)}$$

$$\Delta G_{diff} = \Delta E_{diff} + E_{ZPE} - TS_{vib} \quad \text{(S20)}$$

where ΔE_r represents the reduction energy calculated by Eqn. (S17) and ΔE_{diff} represents the diffusion energy calculated by Eqn. (S18). E_{ZPE} corresponds to the zero-point energy and S_{vib} is the vibrational entropy. If there are n number of N* reduction, we will average the reduction energy by n. If there are n number of N* diffusion, we will average the diffusion energy by n.

The zero-point energy and the temperature dependent vibrational entropy contribution are determined using Eqns. (S21) - (S22), respectively.

$$E_{ZPE} = \frac{1}{2}\sum_i hv_i \quad \text{(S21)}$$

$$S_{vib} = R\sum_i \left[\frac{hv_i}{k_BT} \times \frac{e^{-\frac{hv_i}{k_BT}}}{1-e^{hv_i}} - ln\left(1 - e^{-\frac{hv_i}{k_BT}}\right)\right] \quad \text{(S22)}$$

where v_i represents the vibrational frequency, h is the plank constant, k_B is the Boltzmann constant and R is the gas constant, respectively.

The equilibrium constant of reduction (K_r) and diffusion (K_{diff}) are calculated using Eqns. (S23) - (S24), respectively.

$$K_r = exp\left(\frac{-\Delta G_r}{k_BT}\right) \quad \text{(S23)}$$

$$K_{diff} = exp\left(\frac{-\Delta G_{diff}}{k_BT}\right) \quad \text{(S24)}$$

The equilibrium constants of the diffusion of N^* species at 1/8 ML and 1/4 ML from the surface to the subsurface and from the surface to the bulk as well as the equilibrium constants of reduction per nitrogen of N^* species in the subsurface and in the bulk by hydrogen to form ammonia as a function of temperature is presented in Figure S18.

Table S3. Vibrational frequency of N* species at different locations, namely on the surface of Fe(100), at the subsurface and in the bulk

Nitrogen species	Vibrational frequency (cm^{-1})
N^* at the surface	666, 360, 292
N^* in the subsurface	1319, 946, 101
N^* in the bulk	880, 292, 278

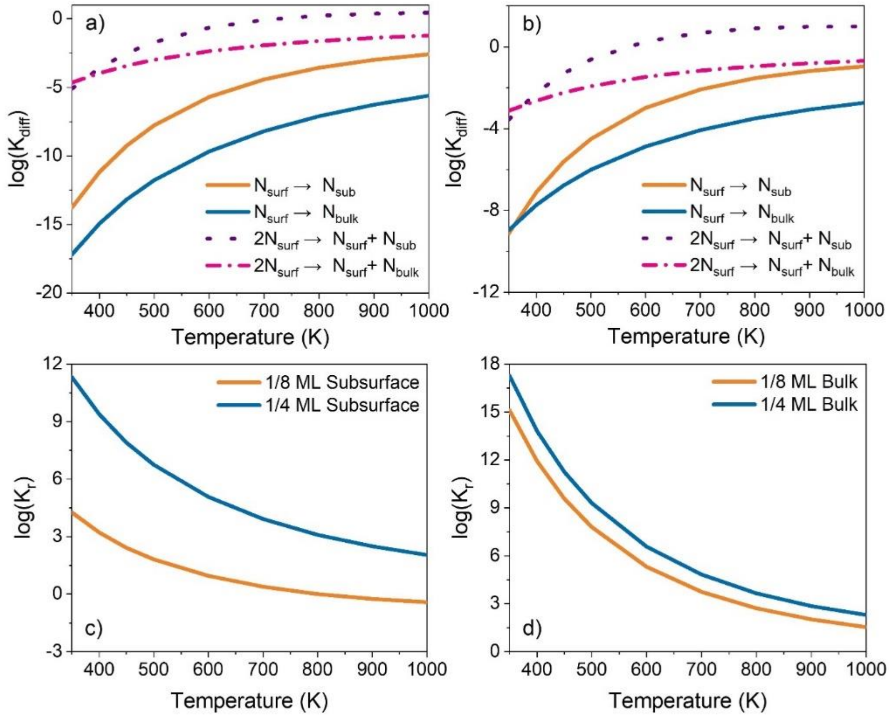

Figure S18. Equilibrium constants of the formation and reduction of N* species within the Fe(100) catalyst as a function of temperature. a) Equilibrium constant of the diffusion per nitrogen of N* species at 1/8 ML from the surface to the subsurface or the bulk in the presence (solid line) and absence (dot line) of pre-adsorbed 1/8 ML N* species. b) Equilibrium constant of the diffusion per nitrogen of N* species at 1/4 ML from the surface to the subsurface or the bulk in the presence (solid line) and absence (dot line) of pre-adsorbed 1/4 ML N* species. c) Equilibrium constants of reduction per nitrogen of N* species in the subsurface by hydrogen to form ammonia at different coverages. d) Equilibrium constants of reduction per nitrogen of N* species in the bulk by hydrogen to form ammonia at different coverages.

References

1. Michalsky, R.; Pfromm, P. H. An Ionicity Rationale to Design Solid Phase Metal Nitride Reactants for Solar Ammonia Production. *J. Phys. Chem. C* **2012**, *116* (44), 23243–23251.
2. O. Levenspiel, *Chemical reaction engineering*, 3rd ed.; Wiley, 1999. 580.
3. Qayyum, A.; Zeb, S.; Naveed, M. A.; Rehman, N. U.; Ghauri, S. A.; Zakaullah, M. Optical Emission Spectroscopy of Ar–N_2 Mixture Plasma. *Journal of Quantitative Spectroscopy and Radiative Transfer* **2007**, *107* (3), 361–371.
4. Qayyum, A.; Zeb, S.; Naveed, M. A.; Ghauri, S. A.; Zakaullah, M.; Waheed, A. Diagnostics of Nitrogen Plasma by Trace Rare-Gas–Optical Emission Spectroscopy. Journal of Applied Physics 2005, 98 (10), 103303.
5. Nakajima, J.; Sekiguchi, H. Synthesis of Ammonia Using Microwave Discharge at Atmospheric Pressure. Thin Solid Films 2008, 516 (13), 4446–4451.
6. Che, F.; Gray, J. T.; Ha, S.; Kruse, N.; Scott, S. L.; McEwen, J.-S., Elucidating the roles of electric fields in catalysis: a perspective. ACS Catalysis 2018, 8 (6), 5153-5174.
7. Che, F.; Zhang, R.; Hensley, A. J.; Ha, S.; McEwen, J.-S., Density functional theory studies of methyl dissociation on a Ni (111) surface in the presence of an external electric field. Physical Chemistry Chemical Physics 2014, 16 (6), 2399-2410.
8. Kresse, G.; Furthmüller, J., Efficient iterative schemes for ab initio total-energy calculations using a plane-wave basis set. Physical review B 1996, 54 (16), 11169.
9. Wan, M.; Yue, H.; Notarangelo, J.; Liu, H.; Che, F., Deep Learning-Assisted Investigation of Electric Field–Dipole Effects on Catalytic Ammonia Synthesis. JACS Au 2022.
10. Kresse, G.; Hafner, J., Norm-conserving and ultrasoft pseudopotentials for first-row and transition elements. Journal of Physics: Condensed Matter 1994, 6 (40), 8245.
11. Davey, W. P., Precision measurements of the lattice constants of twelve common metals. Physical Review 1925, 25 (6), 753.
12. Mortensen, J. J.; Ganduglia-Pirovano, M.; Hansen, L. B.; Hammer, B. Stoltze, P.; Nørskov, J. K., Nitrogen adsorption on Fe (111),(100), and (110) surfaces. Surface science 1999, 422 (1-3), 8-16.
13. Pedersen, M.; Österlund, L.; Mortensen, J. J.; Mavrikakis, M.; Hansen, L. B.; Stensgaard, I.; Lægsgaard, E.; Nørskov, J. K.; Besenbacher, F., Diffusion of N adatoms on the Fe (100) surface. Physical Review Letters 2000, 84 (21), 4898.
14. Che, F.; Gray, J. T.; Ha, S.; McEwen, J.-S., Improving Ni catalysts using electric fields: a DFT and experimental study of the methane steam reforming reaction. ACS Catalysis 2017, 7 (1), 551-562.
